# Magnesium Boryl Reactivity with 9‐BBN and Ph_3_B: Rational B−B′ Bond Formation and Diborane Isomerization

**DOI:** 10.1002/anie.201709902

**Published:** 2017-11-27

**Authors:** Anne‐Frédérique Pécharman, Michael S. Hill, Claire L. McMullin, Mary F. Mahon

**Affiliations:** ^1^ Department of Chemistry University of Bath Bath BA2 7AY UK

**Keywords:** boranes, boryl groups, diborane, magnesium, main-group chemistry

## Abstract

Reactions of a magnesium‐based pinacolatoboryl nucleophile with the electrophilic organoboranes, 9‐BBN and Ph_3_B, provide facile B−B′ single bond formation. Although the Ph_3_B derivative is thermally stable, when heated, the unsymmetrical diborane(5) anion derived from 9‐BBN is found to isomerize to two regioisomeric species via a proposed mechanism involving dehydroboration of the borabicyclo[3.3.1]nonane and syn‐diboration of the resultant alkenyl carbocycle.

The aptitude of carbon for homocatenation is unsurpassed. In contrast, our ability to generate homonuclear E−E bonds between other p‐block elements is at a much more primitive stage of development. Although, for example, boron catenation is common in boron hydride cluster chemistry,^1^ only limited methods are available for the formation of electron‐precise (2c–2e) B−B single bonds.^2^ The generation of B−B bonds within synthetically important diborane(4) molecules such as bis(pinacolato)diborane (B_2_pin_2_), for example, is dependent on the reductive coupling of a haloborane by an alkali metal.^3^ Although there have been recent advances in both the metal‐templated coupling of borylene units^4^ and catalytic^5^ and stoichiometric^2^, ^6^ B−H dehydrocoupling, of more relevance to the current work are Yamashita and Nozaki's reports of the lithium boryltrihydroborate (**1**)^7^ and the triborane(5) derivative (**2**).^8^ In processes reminiscent of classical nucleophilic alkylation reactions, compounds **1** and **2** were synthesized from the nucleophilic lithium boryl anion (**3**)^9^ and the boron electrophiles, BH_3_⋅THF and BF_3_⋅OEt_2_, respectively. 
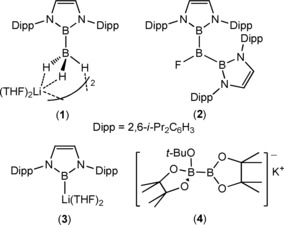


The isolation of the boron nucleophile (**3**) itself, however, requires strongly reducing and problematic reaction conditions and is dependent on the high degree of kinetic stabilization provided by sterically demanding substituents about the boron center.^10^ While diborane adducts such as **4** have also been shown to act as viable surrogates for boron nucleophiles,^11^, ^12^ we have reported that terminal magnesium boryl species may be generated by activation of the B−B bond of B_2_pin_2_ within the coordination sphere of magnesium (Scheme 1).^13^ Treatment of compound **5** with one equivalent of B_2_pin_2_ provided complex **6** in which one of the boron centers of the diborane had been quaternized by addition of the *n*‐butyl group. Addition of further equivalents of B_2_pin_2_ resulted in the displacement of *n*‐BuBpin and the formation of an unusual derivative (**7**) of the catenated triboron [B_3_pin_3_]^−^ anion. Subsequent treatment of **6** or **7** with 2‐dimethylaminopyridine (DMAP) provided the magnesium derivative (**8**) containing a terminal [Bpin]^−^ anion. While compound **8** performs as a well behaved boron centered nucleophile in reactions with both halogenated and non‐halogenated organic electrophiles,^13^ the formation of compound **7** highlights the potential of these systems for the generation of molecules containing electron precise B−B σ bonds. Herein, we extend our study of these magnesium‐centered boron nucleophiles to their reactivity with the organoboranes 9‐borabicyclo[3.3.1]nonane (9‐BBN) and Ph_3_B.

**Scheme 1:**
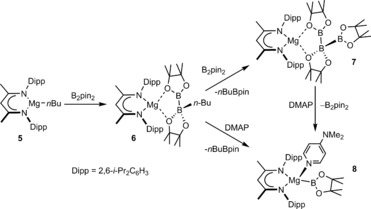
Synthesis of compounds **6**–**8**.

The easily generated [pinB‐Bpin(*n*‐Bu)]^−^ anion of compound **6** is reminiscent of nucleophilic boron surrogates such as **4**. Samples of compound **6** were thus reacted with 0.5 molar equivalents of the 9‐BBN dimer and an equimolar quantity of Ph_3_B. The resultant ^1^H NMR spectra revealed the formation of the β‐diketiminato magnesium compounds, **9** and **10**, for the 9‐BBN‐ and Ph_3_B‐based reactions, respectively (Scheme 2).

**Scheme 2:**
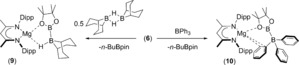
Synthesis of compounds **9** and **10**.

The ^11^B NMR spectra of both reactions provided evidence for the production of *n*‐BuBpin (*δ*=34.2 ppm), while resonances at δ −22.8 ppm (**9**) and −14.6 ppm (**10**) were consistent with the generation of new four‐coordinate boron environments. After removal of volatiles, the *n*‐BuBpin by‐product was readily separated from samples of both **9** and **10** by washing of the colorless solids with *n*‐hexane. In both cases, crystallization from toluene solutions at −35 °C provided samples suitable for single‐crystal X‐ray diffraction analysis (Figure 1). These experiments revealed that both compounds **9** and **10** were magnesium derivatives of unsymmetrical B(sp^2^)−B(sp^3^) anions comprising bonds constructed from a {Bpin} unit and half an equivalent of the 9‐BBN dimer (**9**) and BPh_3_ (**10**), respectively.

**Figure 1:**
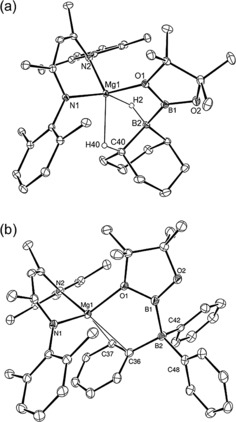
ORTEP representations of a) **9**; b) **10** (ellipsoids set at 25 % probability). Isopropyl methyl groups and hydrogen atoms except H2 and H40 (**9**) are removed for clarity.^18^ Selected bond lengths [Å] and angles [°]: (**9**) Mg1–N1 2.0556(10), Mg1–N2 2.0488(10), Mg1–O1 2.0719(9), B1–B2 1.7203(18); N2‐Mg1‐N1 94.95(4), B1‐O1‐Mg1 93.15(7), O1‐B1‐B2 116.68(10); (**10**) Mg1–O1 2.059(3), Mg–N1 2.043(4), Mg1–N2 2.037(4), Mg1–C36 2.544(5), Mg1–C37 2.410(5), B1–B2 1.718(7), C36–B2 1.647(7), C42–B2 1.649(7), C48–B2 1.630(7); N2‐Mg1‐N1 96.40(14), O1‐B1‐B2 125.2(4), O2‐B1‐O1 108.1(4), O2‐B1‐B2 126.6(4), C36‐B2‐B1 109.3(4), C42‐B2‐B1 106.6(4), C48‐B2‐B1 114.4(4).

The {pinB‐BR_2_X}^−^ anions of both compounds **9** and **10** are reminiscent of the boron‐containing anion of compound **1** and confirm that **6** provides a viable source of the [Bpin]^−^ nucleophile.^7^ The B1−B2 bond lengths (**9**, 1.7203(18); **10**, 1.718(7) Å) are otherwise unremarkable and are comparable to previously reported B(sp^2^)−B(sp^3^) bonds.^12^ The most notable features of compound **9** are the significant interactions of H2 and H40, attached to B2 and C40, respectively, which were located and refined without restraints (Mg1−H2 1.900(15), Mg1−H40 2.220(15) Å), and the magnesium atom. Similarly, the magnesium center of compound **10** displays close contacts between the (C36) *ipso* and the (C37) *ortho* carbons of one of the boron‐bound phenyl substituents (Mg1−C36 2.544(5), Mg1−C37 2.410(5) Å).

We have previously described a number of catalytic systems derived from compound **5** in which pinacolborane is utilized as a reagent for either the hydroboration of polarized multiple bonds or as the hydridic coupling partner during heterodehydrocoupling with organic amines.^14^ In all cases, the liberation of the borylated products was deduced to occur through the assembly of isolable hydridoborate intermediates which undergo subsequent boron‐to‐magnesium hydride elimination as the product‐forming step of the catalysis. To assess the potential for similar hydride or phenyl elimination, samples of **9** and **10** in [D_8_]toluene were heated at 110 °C. Although compound **10** proved to be completely stable, after three days under these conditions the initial sample of **9** had been entirely consumed with the production of two new β‐diketiminato magnesium derivatives, compounds **11** and **12**, in a respective 1:0.7 ratio. Although the ^1^H and ^13^C{^1^H} NMR spectra of the resultant mixture of compounds were otherwise uninformative, the ^11^B NMR spectrum displayed two resonances at δ−14.6 and −19.5 ppm, consistent with the formation of two differentiated four‐coordinate boron environments.

Compounds **11** and **12** were identified by fractional crystallization from a saturated toluene solution at −35 °C and mechanical separation of individual single crystals suitable for X‐ray diffraction analysis (Figure 2). Both compounds crystallized with near identical monoclinic (*P*2_1_/*n*) unit cells and, although neither compound contains a direct B−B bond, were identified to be constitutional isomers of compound **9**. In each case a {Bpin} unit appears to have replaced a C−H bond of a methylene group adjacent to the carbon center attached to the bridgehead boron atom (B2) of the original 9‐BBN reagent. These latter atoms, while still four‐coordinate, now comprise diorganoborohydride residues in both compounds and the structures of **11** and **12** differ only in the constitution of their respective bicyclic [3.3.1] (**11**) and [4.2.1] (**12**) ring systems; whereas the initial six‐membered rings of 9‐BBN are retained in **11**, the bicycle has isomerized to a combination of five‐ and seven‐membered rings in **12**. In both cases the borohydride anion binds to the magnesium center through a combination of O1−Mg1 and B2‐μ_2_‐H_2_‐Mg1 bridging interactions augmented by a further close contact with the C−H bond of the C36‐containing methine unit.

**Figure 2:**
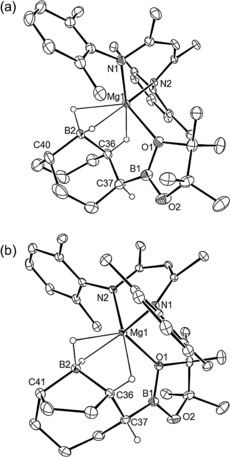
ORTEP representations of a) **11**; b) **12** (ellipsoids set at 25 % probability). Isopropyl methyl groups and hydrogen atoms except those attached to B2, C36, and C37 are removed for clarity.^18^ Selected bond lengths [Å] and angles [°]: (**11**) N1–Mg1 2.066(3), N2–Mg1 2.061(2), O1–B1 1.408(5), O2–B1 1.348(5), C37–B1 1.568(5), C36–B2 1.628(5), C40–B2 1.594(5); N2‐Mg1‐N1 95.40(10), O2‐B1‐O1 111.8(3), C36‐C37‐B1 111.4(3), C40‐B2‐C36 104.9(3); (**12**) N1–Mg1 2.0684(13), N2–Mg1 2.0399(13), O1–B1 1.405(2), O2–B1 1.356(2), C37–B1 1.561(3), C36–B2 1.661(2), C41–B2 1.618(3); N2‐Mg1‐N1 95.67(5), O2‐B1‐O1 111.81(16), C36‐C37‐B1 112.20(14), C41‐B2‐C36 102.24(14).

Yalpani and co‐workers have previously demonstrated that the isomerization of 9‐borabicyclo[3.3.1]nonane to 9‐borabicyclo[4.2.1]nonane may be thermally induced at temperatures >150 °C.^15^ A similar metal‐mediated rearrangement has also been observed during the attempted hydroboration of a zirconocene‐coordinated diphenylacetylene by 9‐BBN.^16^ These earlier reports have suggested such isomerization proceeds through a sequence of de‐hydroboration and intramolecular re‐hydroboration to provide the two possible regioisomers. We thus suggest that the synthesis of compounds **11** and **12** ensues through a similar process and the formation of a common but unobservable borohydride intermediate (**13**), which is the result of thermally induced de‐hydroboration (Scheme 3). Subsequent intramolecular *syn*‐diboration of the alkenyl carbocycle with the unsymmetrical B−B′ bond then takes place to form both of the crystallographically characterized regioisomers, **11** and **12**.

**Scheme 3:**
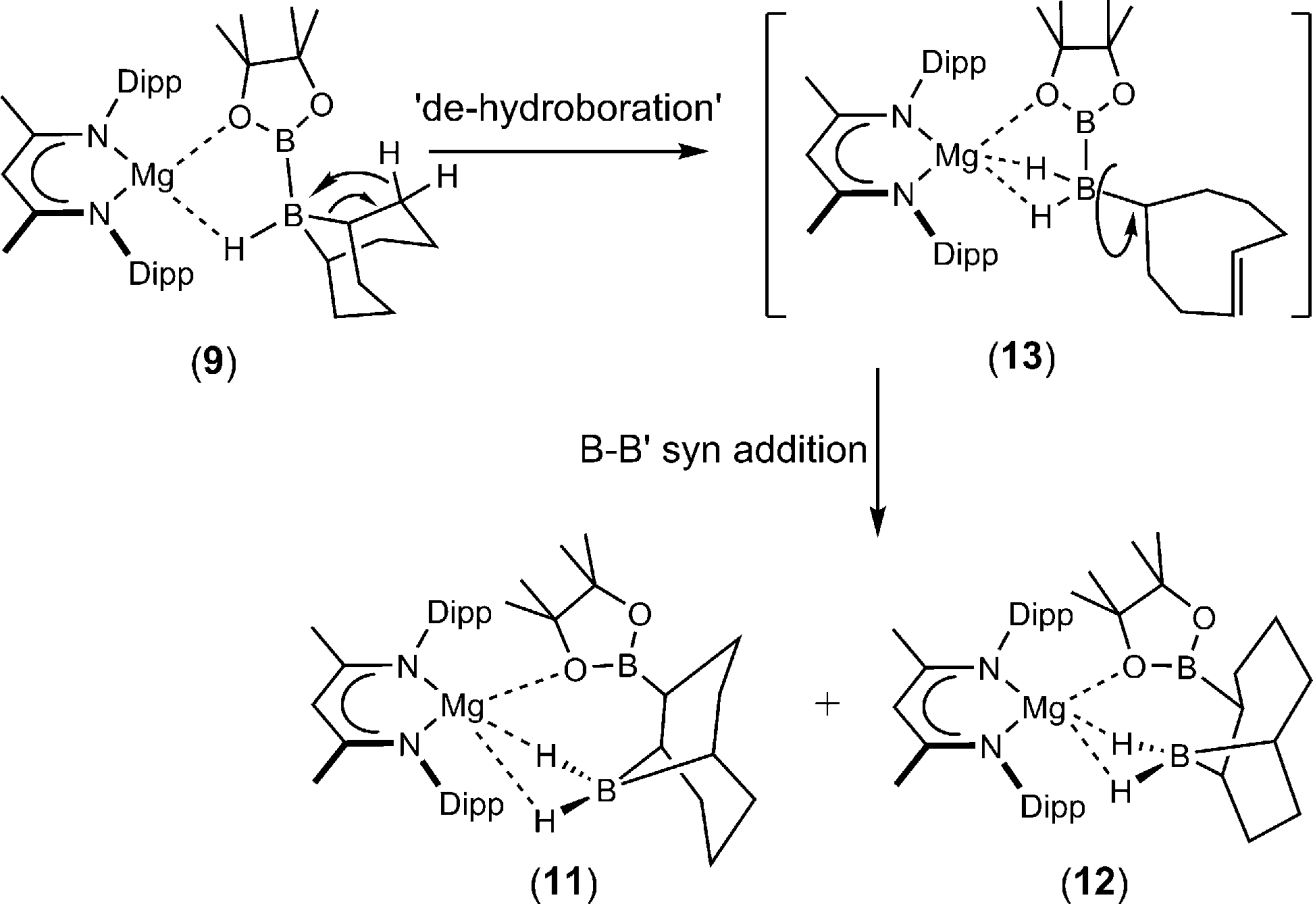
Proposed route to compounds **11** and **12**.

The validity of these proposals was supported by density functional theory (DFT) calculations. Both borohydride derivatives, **11** and **12**, are exergonic relative to structure **9**, with free energies of −6.7 kcal mol^−1^ and −5.1 kcal mol^−1^ respectively, confirming the experimental generation of these species under the applied reaction conditions. The route through intermediate **13** was much more difficult to identify computationally, which is due to the conformational flexibility of the cyclooctenyl ring. A range of intermediates were optimized, ranging in free energy from +19.4 to 31.7 kcal mol^−1^. This conformational freedom also militated against the location of any meaningful transition state for either of the subsequent proposed diboration steps to form compounds **11** and **12**, despite multiple attempts.

In summary, the easily accessed magnesium boryl equivalent **6** reacts with the electrophilic boron centers of 9‐BBN and Ph_3_B to provide anions with electron precise (2c–2e) B−B single bonds. Liberation of the neutral diborane(4) molecule has yet to be achieved. These observations, however, indicate that the reaction of compounds such as **6** with boron reagents containing more labile leaving groups should provide a practicable means to achieve B−B bond formation and facile access to synthetically useful unsymmetrical diboranes.^17^

## Conflict of interest

The authors declare no conflict of interest.

## Supporting information

As a service to our authors and readers, this journal provides supporting information supplied by the authors. Such materials are peer reviewed and may be re‐organized for online delivery, but are not copy‐edited or typeset. Technical support issues arising from supporting information (other than missing files) should be addressed to the authors.

Supplementary
