# Supplementary material for: Magnesium Boryl Reactivity with 9‐BBN and Ph3B: Rational B−B′ Bond Formation and Diborane Isomerization
Source: Angew Chem Int Ed Engl. 2017 Nov 27;56(51):16363–6. doi: 10.1002/anie.201709902 (PMC5767756; doi:10.1002/anie.201709902)
Supplement: Supplementary file 1 — Supplementary [file ANIE-56-16363-s001.pdf]

Angewandte Chemie
Eine Zeitschrift der Gesellschaft Deutscher Chemiker

Supporting Information

Magnesium Boryl Reactivity with 9-BBN and Ph_3B: Rational B−B′ Bond Formation and Diborane Isomerization

Anne-Frédérique Pécharman, Michael S. Hill, Claire L. McMullin,* and Mary F. Mahon*

anie_201709902_sm_miscellaneous_information.pdf

Author Contributions

A.P. Investigation: Equal; Methodology: Equal
C.M. Formal analysis: Equal; Investigation: Equal; Writing—review & editing: Supporting
M.M. Formal analysis: Equal; Investigation: Equal; Writing—review & editing: Supporting.

Experimental Procedures

1. General considerations and starting materials

All manipulations were carried out using standard Schlenk line and glovebox techniques under an inert atmosphere of argon. NMR experiments were conducted in J Young tap NMR tubes made up and sealed in a Glovebox. NMR spectra were collected on a Bruker AV300 spectrometer operating at 300.2 MHz (^{1}H), 75.5 MHz (^{13}C), 96.3 MHz (^{11}B) or an Agilent ProPulse spectrometer operating at 500 MHz (^{1}H), 126 MHz (^{13}C), 160.4 MHz (^{11}B). The spectra were referenced relative to residual solvent resonances or an external $BF_3.OEt_2$ standard (^{11}B). Solvents (toluene, hexane) were dried by passage through a commercially available (Innovative Technologies) solvent purification system, under nitrogen and stored in ampoules over molecular sieves. C_6D_6 and d_8-toluene were purchased from Fluorochem Ltd. and Sigma-Aldrich Ltd. and dried over molten potassium before distilling under argon and storing over molecular sieves. Di-*n*-butylmagnesium (1.0 M solution in *n*-heptane), bis(pinacolato)diborane, 9-BBN and BPh_3 were purchased from Sigma-Aldrich Ltd. and the BPh_3 was sublimed before use. [HC{(Me)CN(2,6-$^{i}Pr_2C_6H_3$)}$_2$Mg*n*Bu] (**5**) and [HC{(Me)CN(2,6-$^{i}Pr_2C_6H_3$)}$_2$Mg{pinBB(*n*-Bu)pin}] (**6**) were synthesized by literature procedures.[1,2] Elemental analysis was carried out at Elemental Microanalysis Ltd., Okehampton, Devon. UK.

2. Synthetic, spectroscopic and analytical data for new compounds

Compound 9

In a J Young NMR tube, d_8-toluene (0.5 mL) was added to a mixture of (**5**) (200 mg, 0.4 mmol) and bis(pinacolato)diboron (107 mg, 0.4 mmol). After 2 hours, 0.5 equivalents of 9-BBN dimer (48.9 mg, 0.2 mmol) were added. After a further 2 hours, the solvent was removed under reduced pressure and the solid was washed with hexane to yield compound **9** (160 mg, 57.8%). Colorless crystals suitable for X-ray diffraction analysis were obtained from a saturated toluene solution at –35˚C. ^{1}H NMR (300 MHz, d_8-tol.): δ 7.12 (m, 6H, Ar-*H*), 4.78 (s, 1H, NC(CH$_3$)C*H*), 3.34 (m, 2H, J_{HH} = 6.9 Hz, C*H*(CH$_3$)$_2$), 3.21 (m, 2H, J_{HH} = 6.9 Hz, C*H*(CH$_3$)$_2$), 1.62 (s, 6H, NC(C*H*$_3$)CH), 2.05-1.40 (m, 13 H, CH$_2$ and CH from 9-BBN), 1.36 (d, 6H, J_{HH} = 6.9 Hz, CH(C*H*$_3$)$_2$), 1.32 (d, 6H, J_{HH} = 6.9 Hz, CH(C*H*$_3$)$_2$), 1.19 (d, 6H, J_{HH} = 6.9 Hz, CH(C*H*$_3$)$_2$), 1.15 (s, 12H, B(OC(C*H*$_3$)$_2$)$_2$), 1.03 (br s, 2H, BH$_2$). ^{13}C{^{1}H} NMR (75 MHz, d_8-tol.): δ 171.0 (N<u>C</u>(CH$_3$)CH), 145.2 (C_{ipso}), 143.1 (C_{ortho}), 143.1 (C_{ortho}), 126.3 (C_{para}), 124.6 (C_{meta}), 124.8 (C_{meta}), 94.7 (NC(CH$_3$)<u>C</u>H), 84.7 (B(O<u>C</u>(CH$_3$)$_2$)$_2$), 36.8 (<u>C</u>H$_2$ 9-BBN), 33.9 (CH$_2$ 9-BBN), 29.2 (<u>C</u>H(CH$_3$)$_2$), 28.4 (<u>C</u>H(CH$_3$)$_2$), 26.4 (B(OC(<u>C</u>H$_3$)$_2$)$_2$), 26.3 (B(OC(<u>C</u>H$_3$)$_2$)$_2$), 26.1 (CH(<u>C</u>H$_3$)$_2$), 25.6 (CH(<u>C</u>H$_3$)$_2$), 25.3 (9-BBN), 25.3 (9-BBN), 25.2 (NC(<u>C</u>H$_3$)). ^{11}B{^{1}H} NMR (96 MHz, d_8-tol): δ –22.8 ppm. Elemental analysis: Found C, 74.46; H, 9.64; N, 4.09 %. $C_{43}H_{68}B_2MgN_2O_2$ requires: C, 74.75; H, 9.92; N, 4.05 %.

Compound 10

In a J Young NMR tube, d$_8$-toluene (0.5 mL) was added to a mixture of compound (**5**) (50 mg, 0.1 mmol) and bis(pinacolato)diboron (25.4 mg, 0.1 mmol). After 2 hours, an equimolar equivalent of triphenylborane (24.2 mg, 0.1 mmol) was added. The solution was left at room temperature for 2 days. The solvent was removed under reduced pressure and the solid was washed with hexane to yield compound **10** as a colorless solid (50 mg, 61%) Colorless crystals suitable for X-ray diffraction analysis were obtained from a saturated hexane solution at –35˚C. ^{1}H NMR (500 MHz, d$_8$-tol.) δ 7.84 (m, 4H, C*H* Ar), 7.67 (m, 2H, C*H* Ar), 7.21 (m, 4H, C*H* Ar), 7.07 (m, 6H, C*H* Ar), 6.99 (m, 3H, C*H* Ar), 6.85 (m, 1H, C*H* Ar), 6.40 (m, 2H, C*H* Ar), 4.58 (s, 1H, NC(CH$_3$)C*H*), 2.90 (m, 2H, C*H*(CH$_3$)$_2$), 2.71 (m, 2H, C*H*(CH$_3$)$_2$), 1.42 (s, 6H, NC(CH_3)CH), 1.30 (m, 12H, CH_3), 1.25 (s, 6H, CH_3), 1.04 (d, 6H, CH_3), 1.00 (m, 12H, CH_3) ppm. ^{13}C{^{1}H} NMR (126 MHz, d$_8$-tol.) δ 170.6 (N*C*(CH$_3$)CH), 144.8, 142.7, 142.0, 134.70 (*C*H Ar), 133.8 (*C*H Ar), 131.4 (*C*H Ar, correlated with resonance at 6.40 ppm in ^{1}H NMR by HSQC), 126.7 (*C*H Ar), 125.9 (*C*H Ar, correlated with resonance at 6.85 ppm in ^{1}H NMR by HSQC), 124.3 (*C*H Ar), 124.1 (*C*H Ar), 123.0 (*C*H Ar), 95.8 (NC(CH$_3$)*C*H), 88.9 (B(O*C*(CH$_3$)$_2$)$_2$), 81.6 (B(O*C*(CH$_3$)$_2$)$_2$), 28.0 (*C*H(CH$_3$)$_2$), 27.5 (*C*H(CH$_3$)$_2$), 26.3 (*C*H$_3$), 25.5 (NC(*C*H$_3$)CH), 25.4 (*C*H$_3$), 25.1 (*C*H$_3$), 24.5 (*C*H$_3$), 23.7 (*C*H$_3$) ppm. ^{11}B NMR (160 MHz, d$_8$-tol.) δ –14.6 ppm. Despite multiple attempts an accurate microanalysis could not be obtained for this compound.

Compounds 11 and 12

A solution of compound **9** was heated in d$_8$-toluene at 110˚C for 3 days. Analysis by ^{1}H and ^{11}B NMR spectroscopy at this point indicated the formation of two new compounds, **11** and **12** in an approximate 1:0.7 ratio. Crystallization from the reaction solution provided a mixture of crystals of both compounds suitable for X-ray diffraction analysis. Removal of volatiles from the reaction mixture and washing of the resultant colorless solid with *n*-hexane effected separation of compound **11** to leave a solid sample that displayed only ^{1}H NMR resonances associated with compound **12**. The *n*-hexane filtrate was dried under vacuum and redissolved in d$_8$-toluene. The ^{1}H and ^{11}B spectra displayed resonances only for compound **11**. (**11**): ^{1}H NMR (500 MHz, d$_8$-tol.) δ 7.08 (m, aromatic CH), 4.89 (s, 1H, NC(CH$_3$)C*H*), 3.12 (m, 2H, J_{HH} = 6.3 Hz, C*H*(CH$_3$)$_2$), 1.66 (s, 6H, NC(CH_3)CH), 1.34 (d, 12H, J_{HH} = 6.3 Hz, CH(CH_3)$_2$), 1.15 (d, 12H, J_{HH} = 6.3 Hz, CH(CH_3)$_2$), 1.08 (s, 12H, B(OC(CH_3)$_2$)$_2$) ppm (other CH$_2$ and CH from 9-BBN in the baseline). ^{13}C NMR (126 MHz, d$_8$-tol.) δ 170.2 (N$\underline{C}$(CH$_3$)CH), 141.6, 135.9, 126.0 (*C*H Ar), 123.8 (*C*H Ar), 123.6 (*C*H Ar), 122.9 (*C*H Ar), 94.9 (NC(CH$_3$)$\underline{C}$H), 81.6 (B(O$\underline{C}$(CH$_3$)$_2$)$_2$), 35.1, 33.2, 28.5 ($\underline{C}$H(CH$_3$)$_2$), 24.5 (B(OC($\underline{C}$H$_3$)$_2$)$_2$), 23.9 ($\underline{C}$H$_3$), 23.8 ($\underline{C}$H$_3$), 23.3 (NC($\underline{C}$H$_3$)) ppm. ^{11}B NMR (160 MHz, d$_8$-tol.) δ –17.7 ppm. (**12**) ^{1}H NMR (500 MHz, d$_8$-tol.) δ 7.13- 6.99 (m, aromatic CH), 4.85 (s, 1H , NC(CH$_3$)C*H*), 3.47 (m, 2H, J_{HH} = 6.7 Hz, C*H*(CH$_3$)$_2$), 3.23 (m, 2H, J_{HH} = 6.7 Hz, C*H*(CH$_3$)$_2$), 1.63 (s, 6H, NC(CH_3)CH), 1.45 (d, 6H, J_{HH} = 6.7 Hz, CH(CH_3)$_2$), 1.20 (m, 34H, CH(CH_3)$_2$ + B(OC(CH_3)$_2$)$_2$ + CH$_2$), 1.08 (s, 2H, BH$_2$) (other CH$_2$ and CH from 9-BBN in the baseline). ^{13}C NMR (126 MHz, d$_8$-tol.) δ 170.3

(N*C*(CH_3)CH), 146.4, 144.6, 142.7, 142.4, 141.6, 135.9, 125.3 (*C*H Ar), 124.1(*C*H Ar), 124.0 (*C*H Ar), 123.8 (*C*H Ar), 123.6 (*C*H Ar), 122.9 (*C*H Ar), 95.4 (NC(CH_3)*C*H), 85.8 (B(O*C*(CH_3)$_2$)$_2$), 34.7, 32.5, 32.4, 31.6, 28.2 (*C*H(CH_3)$_2$), 27.8 (*C*H(CH_3)$_2$), 25.8, 25.7, 25.3, 24.5 (NC(*C*H_3)), 24.4 (NC(*C*H_3)), 24.2, 23.3, 22.7, 22.0. ^{11}B NMR (160 MHz, d_8-tol.) δ –22.5.

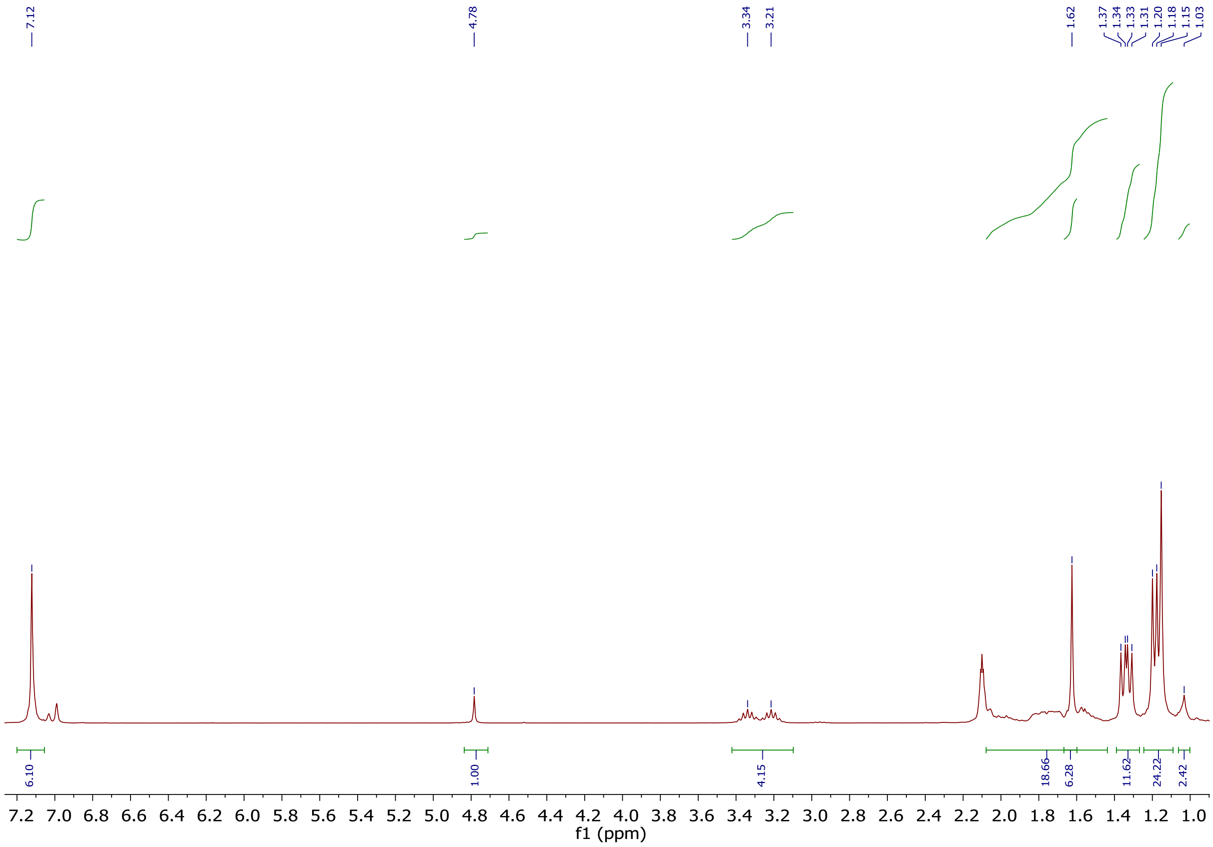

Figure S1: ^{1}H NMR (500 MHz) spectrum of compound **9**.

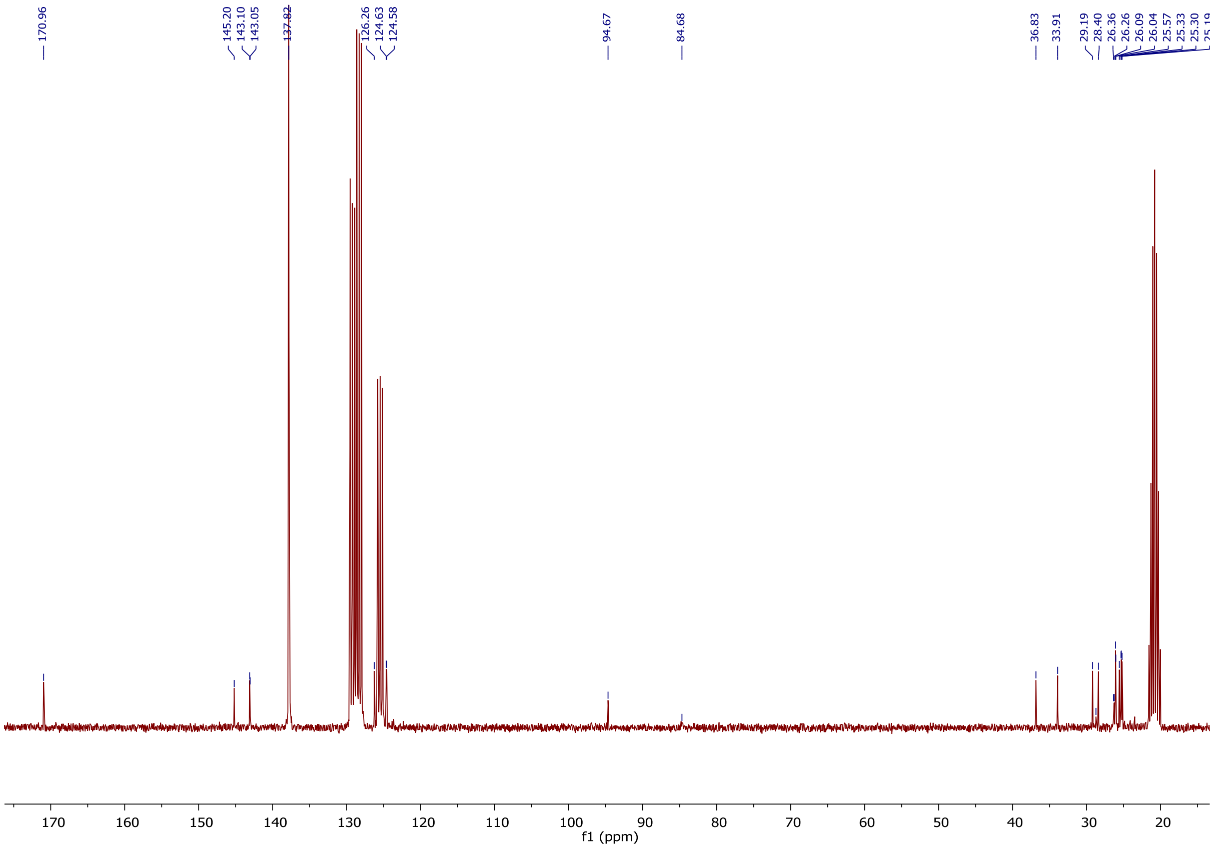

Figure S2: $^{13}C\{^1H\}$ NMR spectrum (126 MHz) of compound **9**.

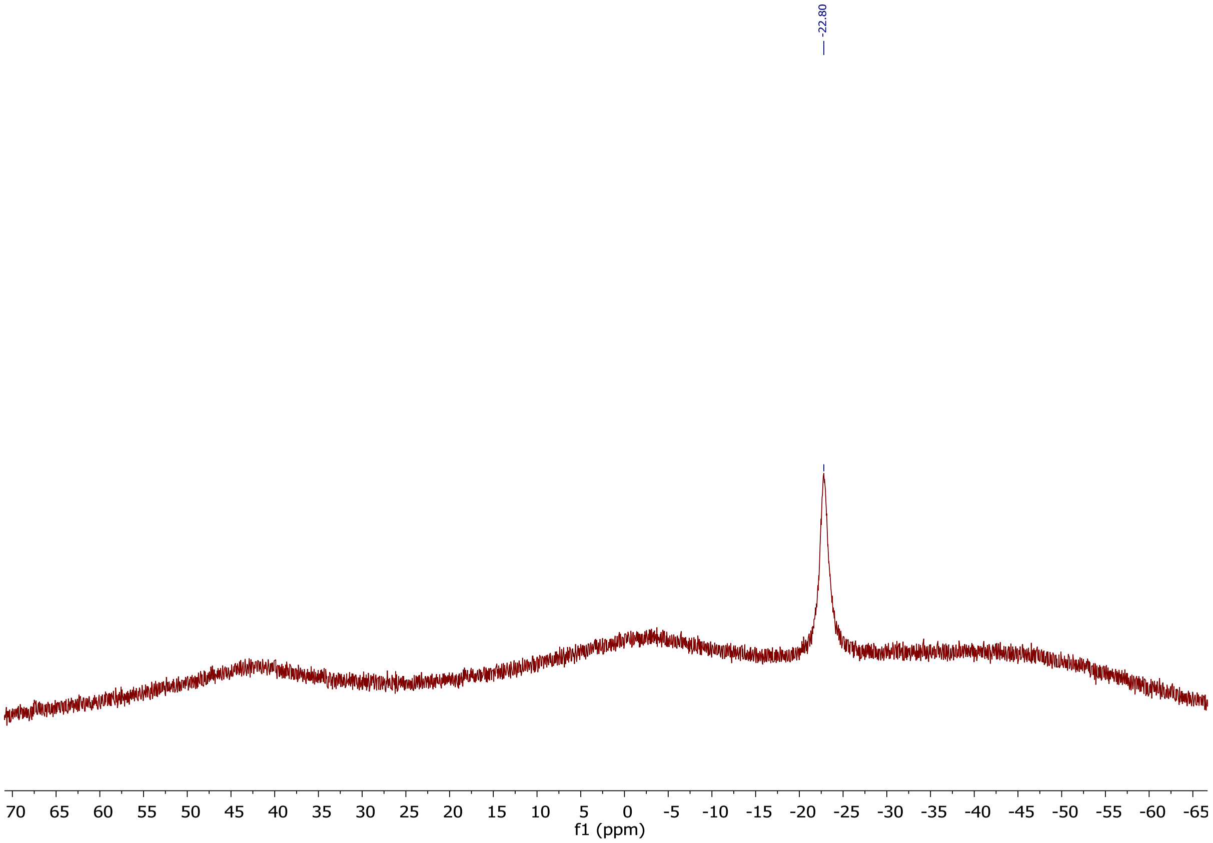

Figure S3: ^{11}B NMR spectrum (160.4 Mz) of compound **9**.

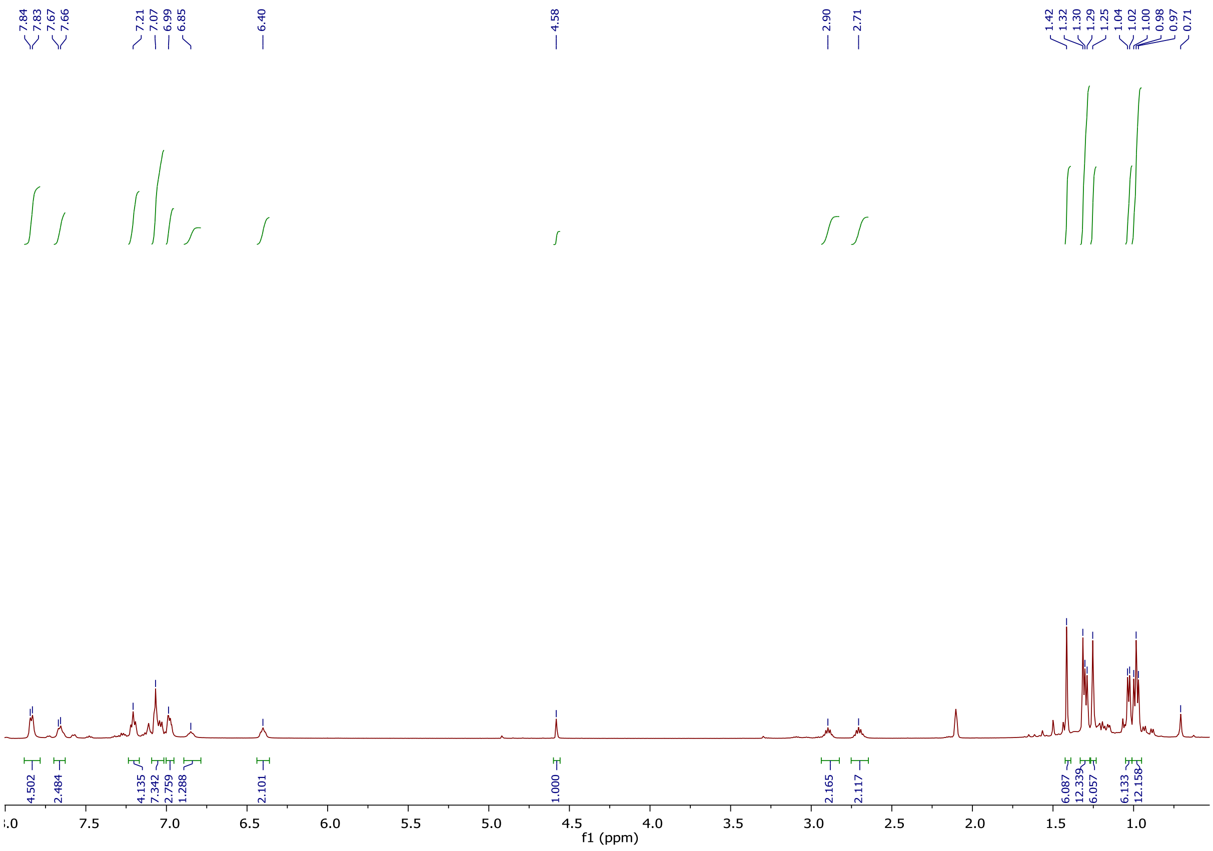

Figure S4: ^{1}H NMR (500 MHz) spectrum of compound **10**.

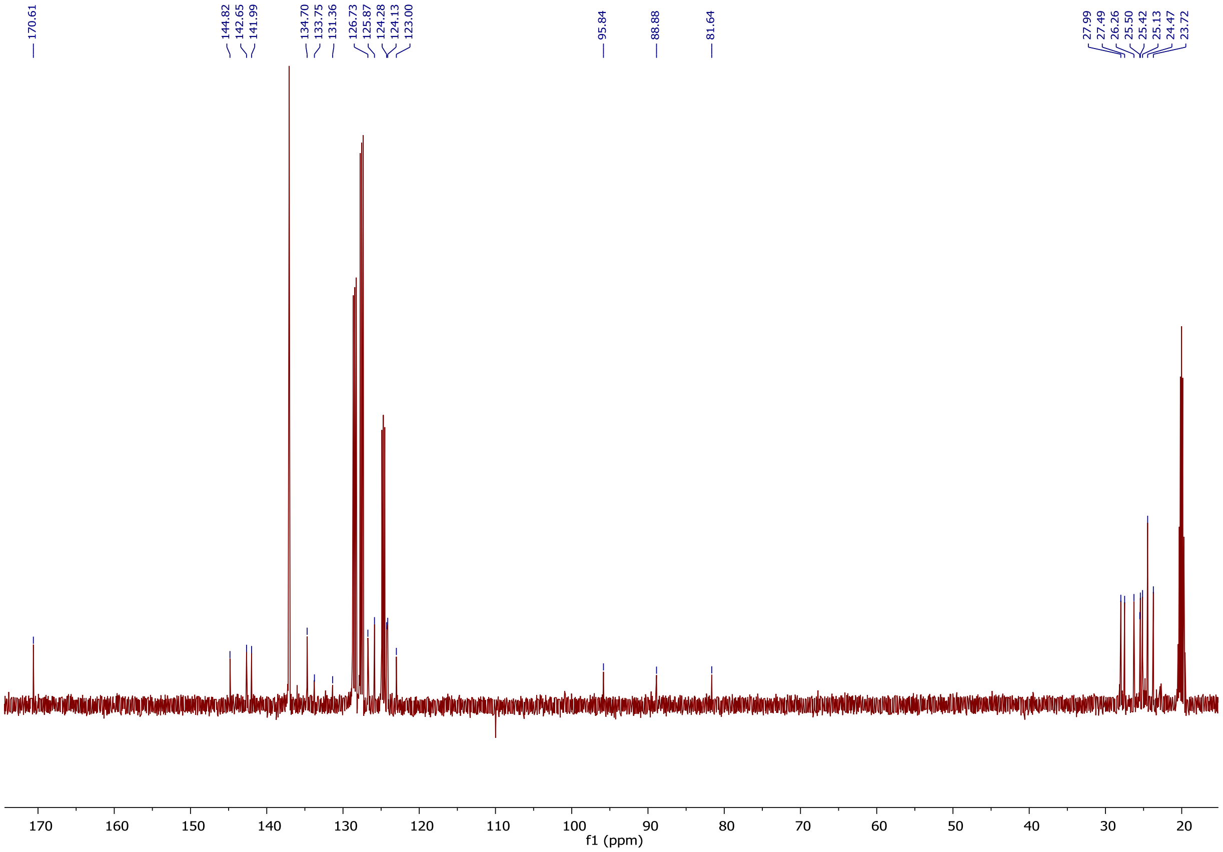

Figure S5: ^{13}C{^{1}H} NMR (126 MHz) spectrum of compound **10**.

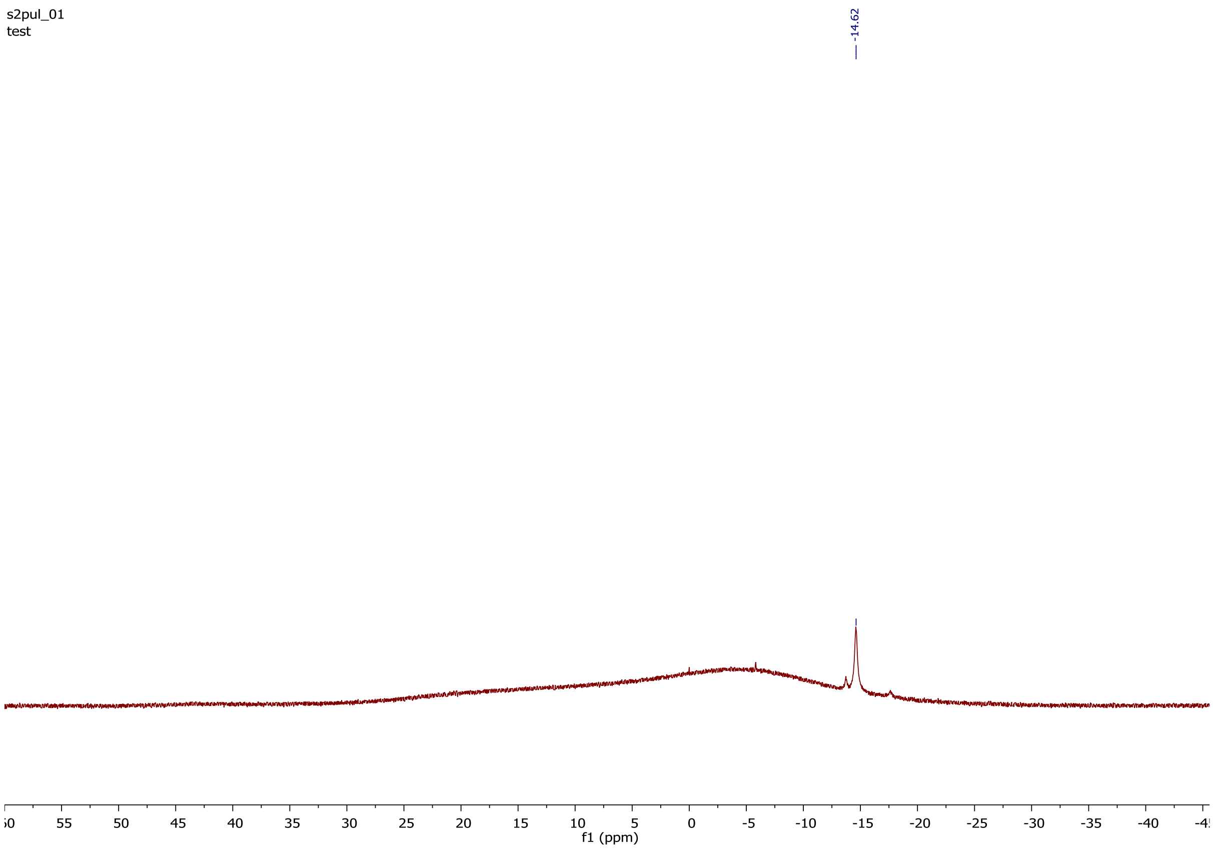

Figure S6: ^{11}B NMR (160.4 MHz) spectrum of compound **10**.

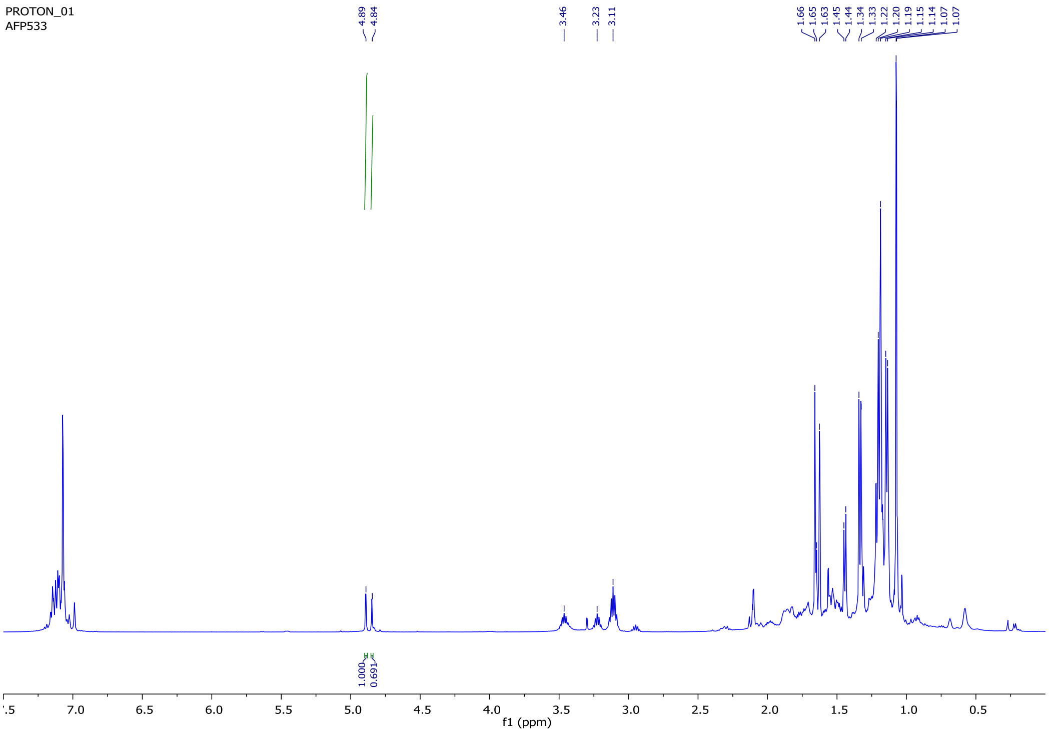

Figure S7: ^{1}H NMR (500 MHz) spectrum of a mixture of compounds **11** and **12** after heating a sample of compound **9** at 110 °C for 4 days.

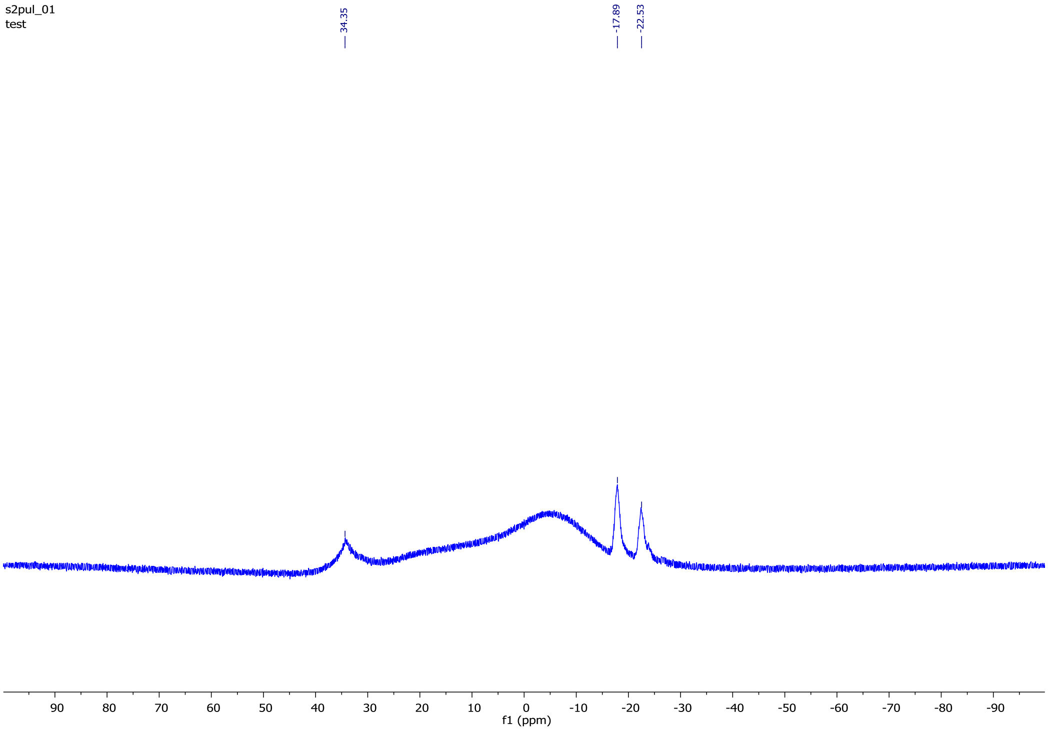

Figure S8: ^{11}B NMR (160.4 MHz) spectrum of a mixture of compounds **11** and **12** after heating a sample of compound **9** at 110 °C for 4 days.

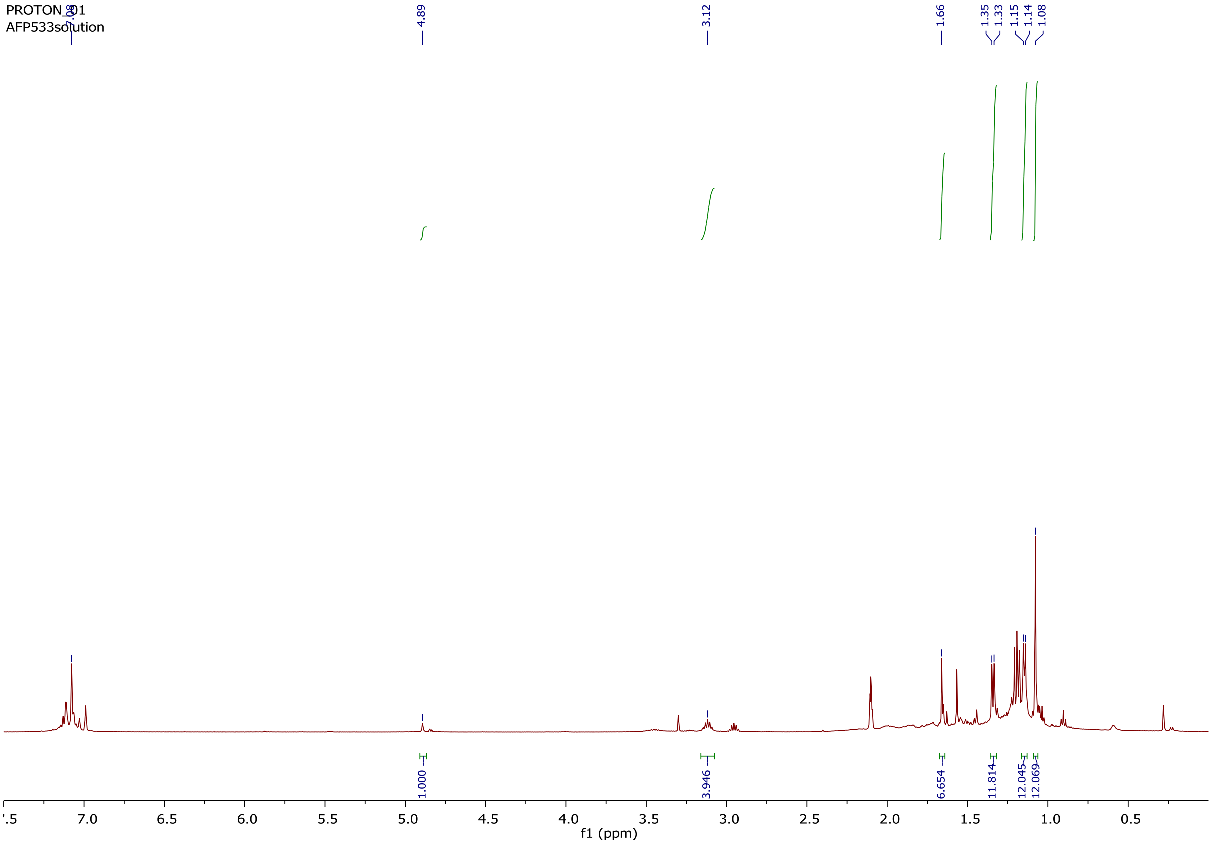

Figure S9: ^{1}H NMR (500 MHz) spectrum of compound **11**.

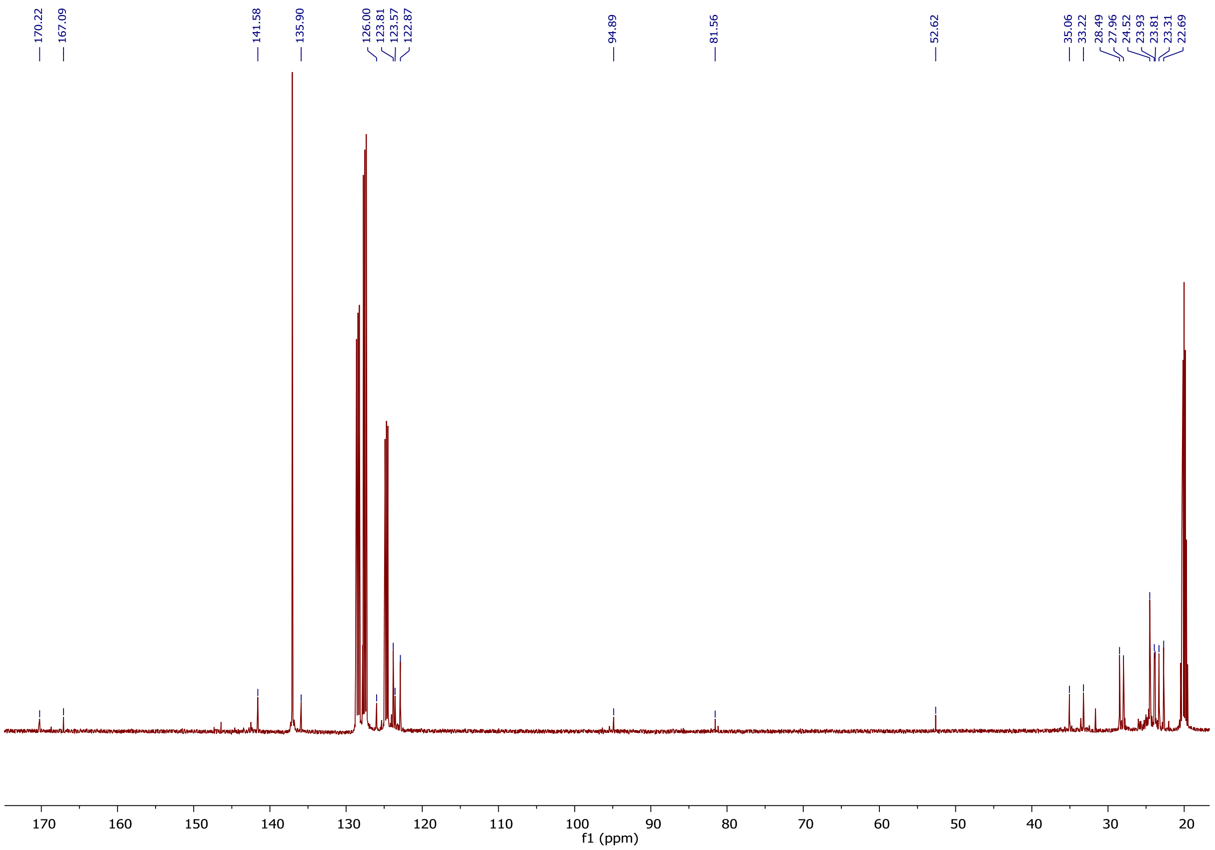

Figure S10: $^{13}C\{^{1}H\}$ NMR (126 MHz) spectrum of compound **11**.

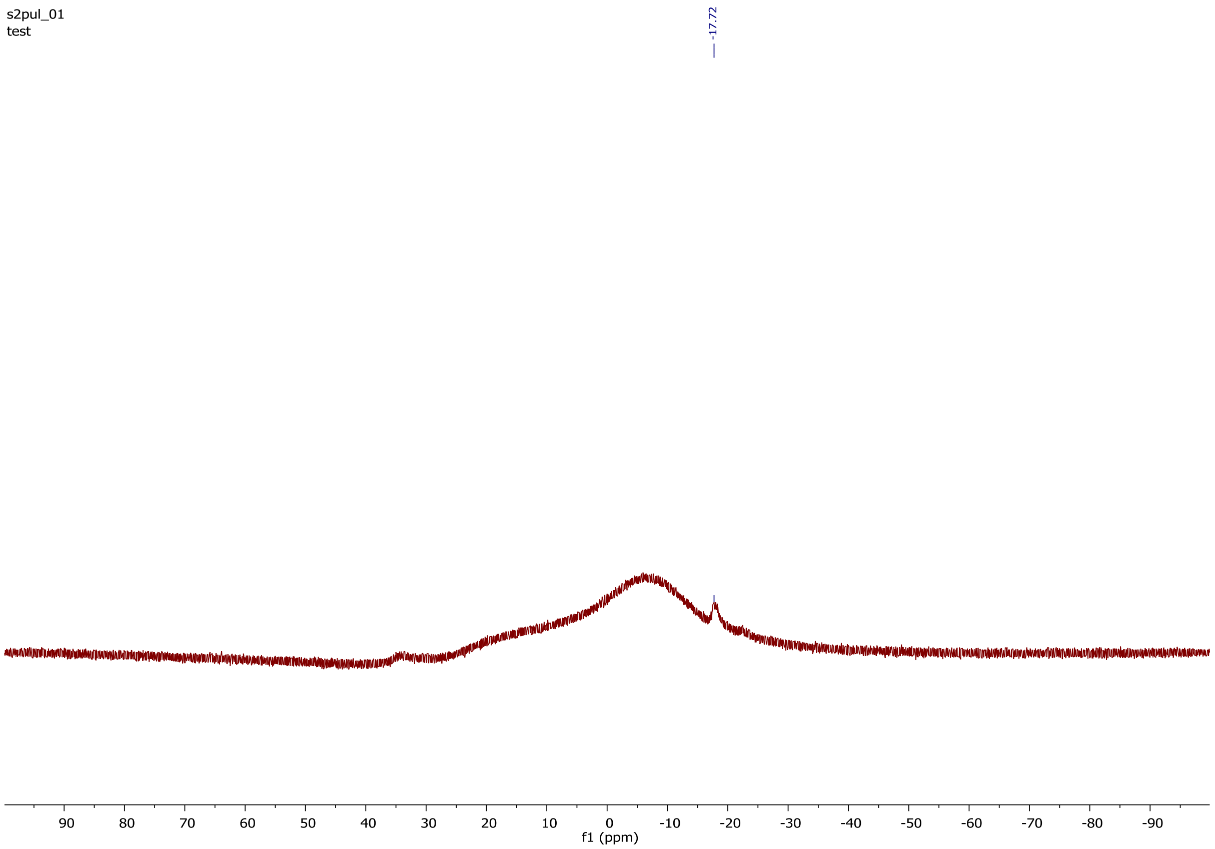

Figure S11: ^{11}B NMR (160.4 MHz) spectrum of compound **11**.

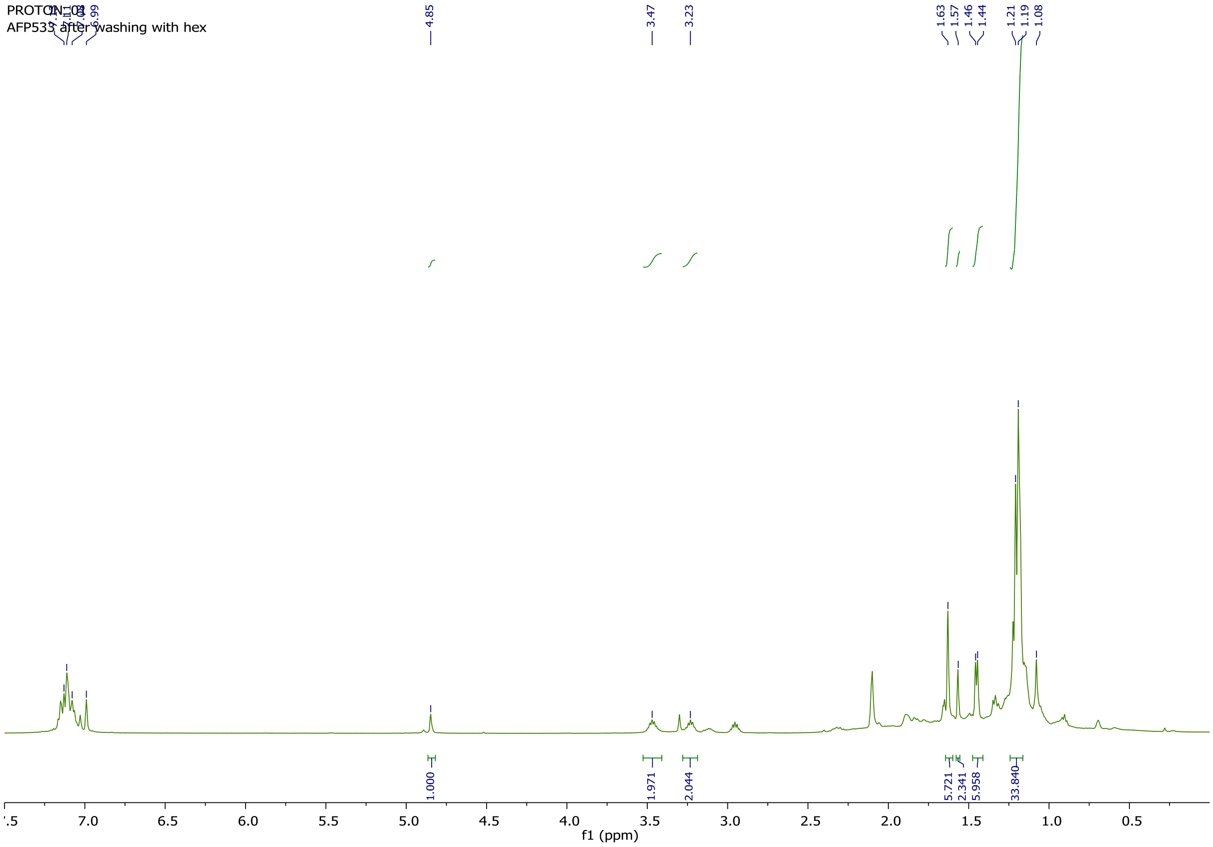

Figure S12: ^{1}H NMR (500 MHz) spectrum of **12**.

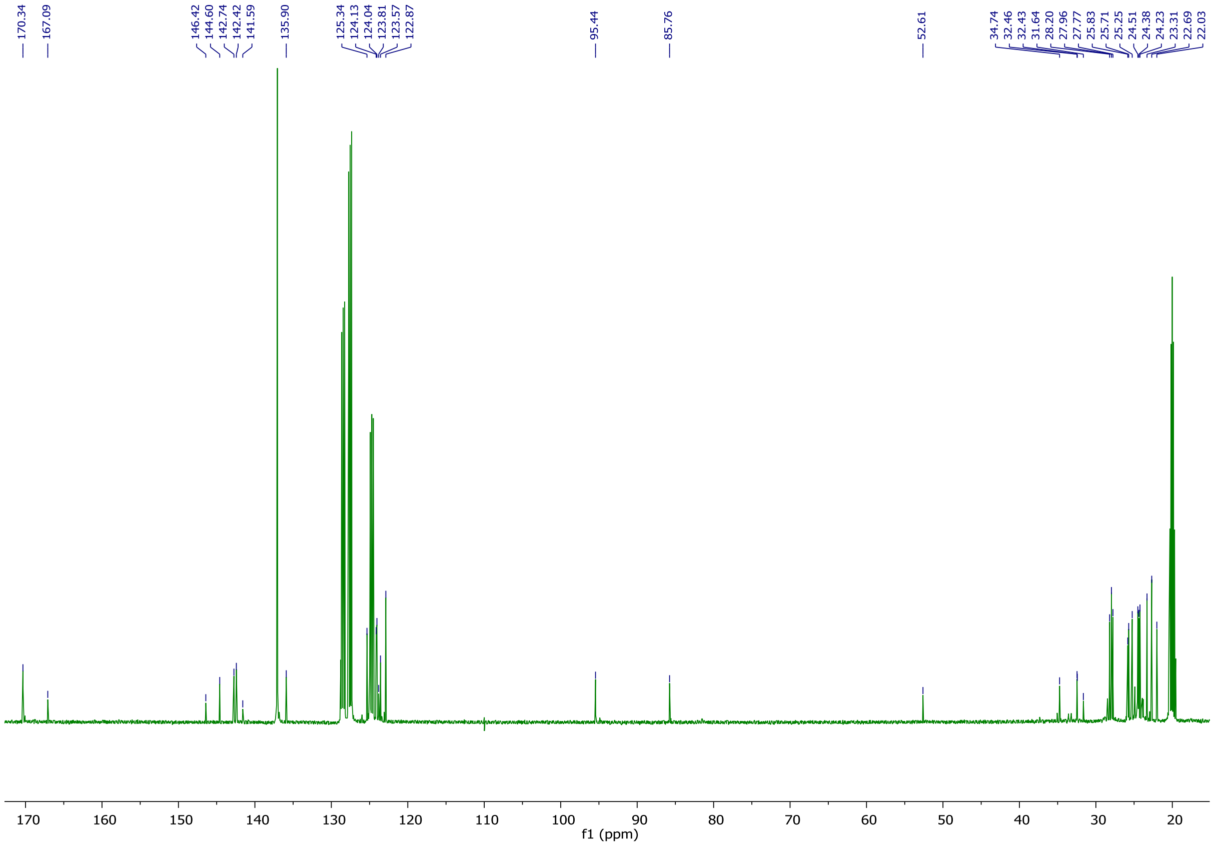

Figure S13: $^{13}C\{^{1}H\}$ NMR (126 MHz) spectrum of compound **12**.

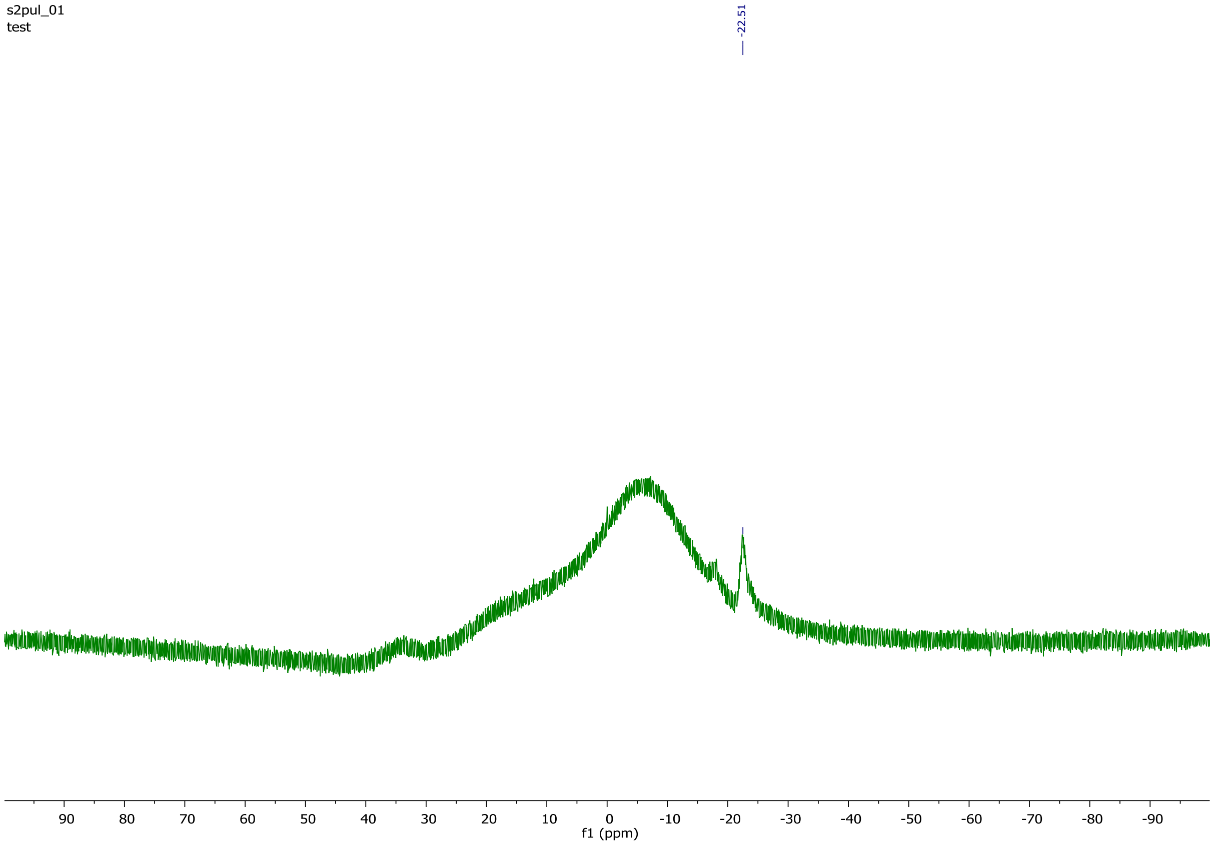

Figure S14: ^{11}B NMR (160.4 MHz) NMR spectrum of compound **12**.

Computational Details / Methodology

DFT calculations were run with Gaussian 09 (Revision D.01).[3] Mg centers were described with the Stuttgart RECPs and associated basis sets,[4] and 6-31G** basis sets were used for all other atoms (BS1).[5] Initial BP86[6] optimizations were performed using the 'grid = ultrafine' option, with all stationary points being fully characterized via analytical frequency calculations as either minima (all positive eigenvalues) or transition states (one negative eigenvalue). All energies were recomputed with a larger basis set (BS2) featuring 6-311++G** on all atoms. Corrections for the effect of toluene (ε = 2.3741) solvent were run using the polarizable continuum model and BS1.[7] Single-point dispersion corrections to the BP86 results employed Grimme's D3 parameter set with Becke-Johnson damping as implemented in Gaussian.[8]

Breakdown of Energy Contributions

The following tables detail the evolution of the relative energies as the successive corrections to the initial SCF energy are included. Terms used are:

**ΔE_{BS1}**	SCF energy computed with the BP86 functional with BS1
**ΔH_{BS1}**	Enthalpy at 0 K with BS1
**ΔG_{BS1}**	Free energy at 298.15 K and 1 atm with BS1
**$\Delta G_{BS1/tol}$**	Free energy corrected for toluene solvent with BS1
**$\Delta G_{BS1/tol+D3}$**	Free energy corrected for toluene and dispersion effects with BS1
**ΔG_{tol}**	Free energy corrected for basis set (BS2), dispersion effects and toluene solvent

Energy Tables

Table S1 – Computed relative energies (kcal/mol) for the reaction of complex **9**. Data in bold are those used in the main text. All energies are quoted relative to **9** at 0.0 kcal/mol.

	**ΔE_{BS1}**	**ΔH_{BS1}**	**ΔG_{BS1}**	**$\Delta G_{BS1/tol}$**	**$\Delta G_{BS1/tol+D3}$**	**ΔE_{BS2}**	**ΔG_{tol}**
**9**	0.0	0.0	0.0	0.0	0.0	0.0	**0.0**
**9_{Xray}**	0.0	0.1	0.3	-0.3	3.7	0.2	**3.8**
**11**	-6.5	-6.7	-3.6	-3.1	-6.9	-6.4	**-6.7**
**12**	-4.4	-4.8	-1.8	-1.1	-5.0	-4.5	**-5.1**
**13a**	18.8	16.2	14.7	14.4	22.1	16.1	**19.4**
**13b**	23.1	20.8	20.3	20.3	24.6	21.0	**22.5**
**13c**	23.7	21.4	20.8	20.7	25.0	21.4	**22.8**
**13d**	33.0	29.9	28.2	27.9	34.2	30.5	**31.7**

Computational Structural Discussion

Two different conformers of **9**, the unsymmetrical diborane anion complex with a {Bpin} and a 9-BBN unit, have been computationally optimised. The X-ray crystal structure, $\mathbf{9_{Xray}}$, is slightly raised in free energy by 3.8 kcal mol^{-1}, compared to the computationally built geometry **9**. Comparison of the structures by overlaying the two geometries (mapping the equivalent Mg centres, nacnac ligands and B atoms to be aligned), shows the majority of misalignment is due to the 9-BBN unit position (Figure S1). Upon closer inspection the conformational difference can be best described by the torsion O-B_{Bpin}-B_{9BBN}-H (shown in red in Figure S2), which is ~ -5° for the crystal structure geometry ($\mathbf{9_{Xray}}$, magenta) and ~ 47° for the computationally optimised structure **9** (shown in blue / elemental colours).

Looking at the breakdown in energy corrections for the two structures, the difference in $\mathbf{\Delta G_{tol}}$ arises from dispersion contributions, with the computed **9** involving more favourable and closer contacts between the 9-BBN unit and the Dipp groups of the nacnac ligand in comparison to the solid state structure $\mathbf{9_{Xray}}$.

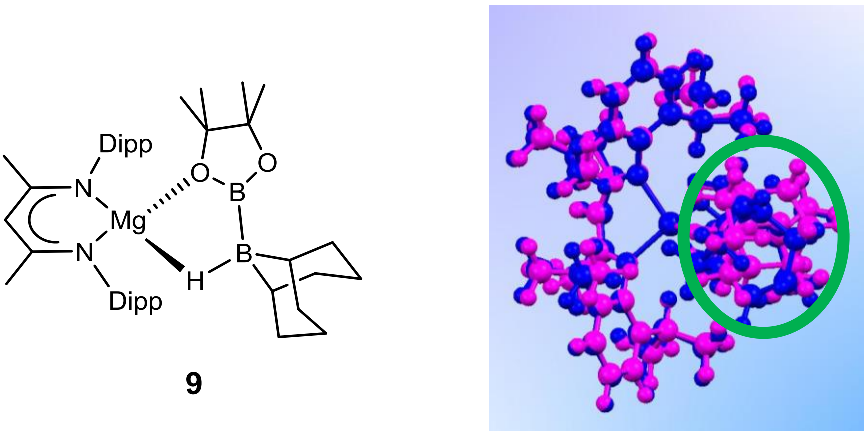

Figure S1 – Overlay of **9** (blue) and $\mathbf{9_{Xray}}$ (magenta) in Mercury, with the unaligned part of the structure (9-BBN unit) circled in green

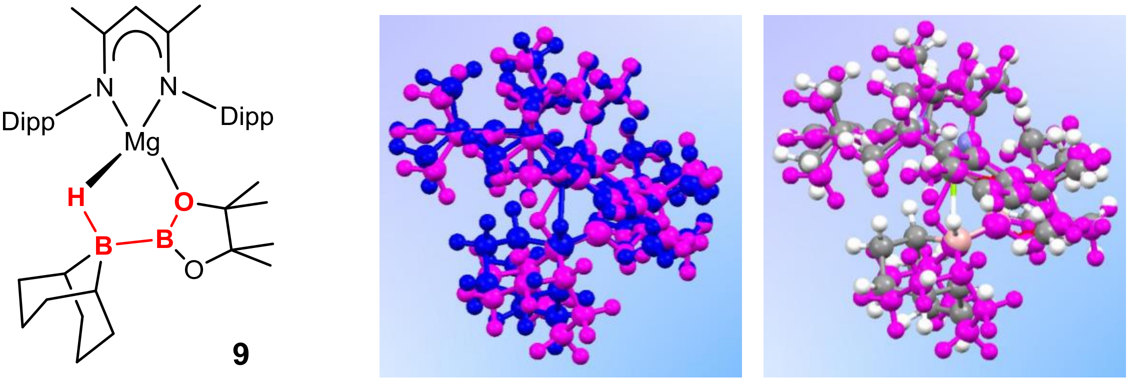

Figure S2 – Overlay of **9** (blue / elemental colours) and $\mathbf{9_{Xray}}$ (magenta) in Mercury

Four intermediates of **13** were optimised (see Figure S3) highlighting the incredible conformational freedom the cyclooctenyl ring can access after “de-hydroboration”.

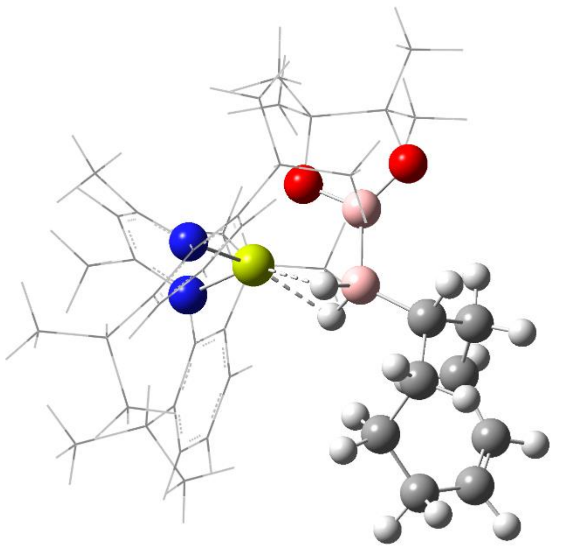

13a

+19.4 kcal mol^{-1}

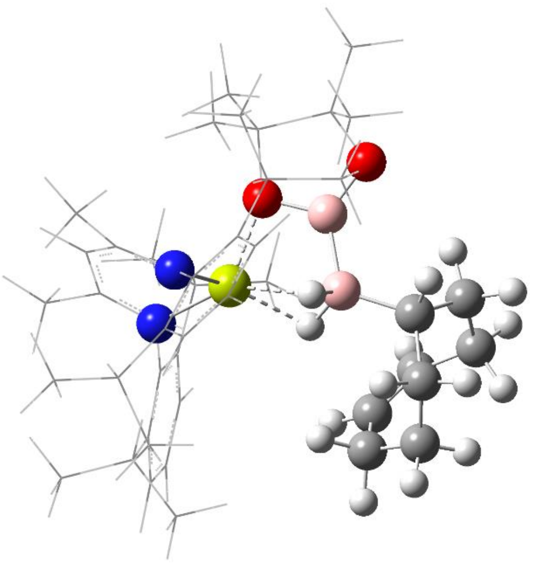

13b

+22.5 kcal mol^{-1}

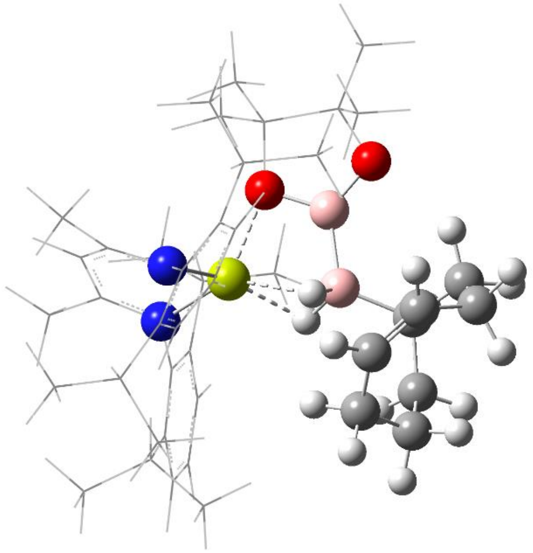

13c

+22.8 kcal mol^{-1}

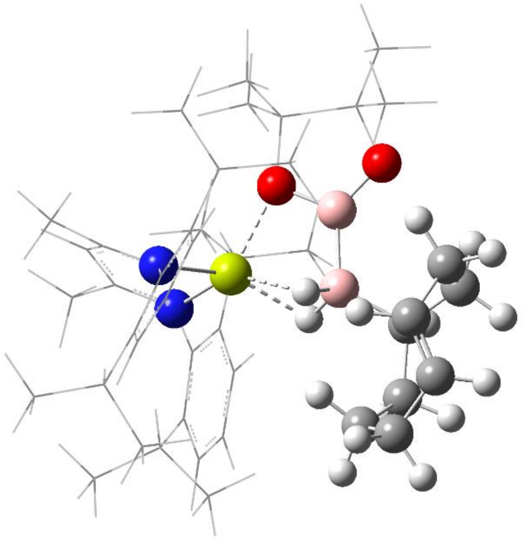

13d

+31.7 kcal mol^{-1}

Figure S3 – DFT-computed geometries for the addition of MeI to complex **11**. Bond lengths given in Ångstroms.

Cartesian Coordinates and Computed Energies (in Hartrees)

9 (comp)
SCF (BP86) Energy = -1990.21226964
Enthalpy 0K = -1989.202017
Enthalpy 298K = -1989.144188
Free Energy 298K = -1989.293198
Lowest Frequency = 6.6931 cm^{-1}
Second Frequency = 17.1422 cm^{-1}
SCF (Toluene) Energy = -1990.21787291
SCF (BP86-D3BJ) Energy = -1990.51277581
SCF (BS2) Energy = -2189.93124956

Mg	0.05635	0.09441	0.16354
O	-1.14003	1.69919	0.88674
O	-1.83794	3.52411	-0.32793
N	-0.59040	-1.72100	0.98409
N	1.98407	0.01463	0.95480
C	-0.64279	-3.06317	3.10722
H	-0.93091	-2.64945	4.08887
H	0.14034	-3.81645	3.29941
H	-1.50964	-3.57209	2.66674
C	-0.09534	-1.94672	2.22067
C	0.97586	-1.21843	2.81286
H	1.15024	-1.47128	3.86317
C	2.01591	-0.45314	2.22132
C	3.23922	-0.21663	3.09753
H	4.00284	-0.99401	2.92212
H	2.96551	-0.25705	4.16327
H	3.71344	0.75222	2.87811
C	3.22483	0.45236	0.35498
C	3.51366	1.84012	0.20212
C	4.72202	2.22923	-0.40785
H	4.94055	3.29745	-0.51657
C	5.64686	1.28719	-0.86646
H	6.58273	1.60883	-1.33531
C	5.35624	-0.07270	-0.72184
H	6.07259	-0.81690	-1.08802
C	4.16104	-0.51542	-0.12245
C	3.91027	-2.02349	-0.05672
H	2.94882	-2.19079	0.45848
C	3.79336	-2.61025	-1.48197
H	2.97546	-2.13459	-2.04808
H	3.60276	-3.69695	-1.44117
H	4.72595	-2.45775	-2.05279
C	5.00850	-2.77303	0.73275
H	5.98920	-2.69625	0.23109
H	4.76098	-3.84597	0.81354
H	5.12981	-2.37604	1.75461
C	2.57723	2.92955	0.71505
H	1.67072	2.42415	1.08802
C	2.15382	3.90505	-0.40446
H	3.01920	4.46426	-0.80151
H	1.43228	4.64997	-0.02347
H	1.67875	3.37035	-1.24223
C	3.20778	3.70093	1.89836
H	3.47027	3.03000	2.73399
H	2.51290	4.46798	2.28444
H	4.13122	4.21866	1.58461
C	-1.55403	-2.65773	0.43950
C	-2.94849	-2.50456	0.70021
C	-3.84880	-3.45695	0.18270
H	-4.91786	-3.34782	0.39664
C	-3.40946	-4.53512	-0.58965
H	-4.12422	-5.26858	-0.97730
C	-2.04548	-4.65832	-0.87101
H	-1.69968	-5.49434	-1.48718
C	-1.10148	-3.73558	-0.37930
C	0.37546	-3.94524	-0.71630
H	0.89537	-2.98605	-0.53862
C	0.59052	-4.33907	-2.19420
H	0.19338	-5.34563	-2.41339
H	1.66674	-4.35999	-2.43126
H	0.10208	-3.62527	-2.87730
C	1.01444	-5.00089	0.21702
H	0.94097	-4.70747	1.27672
H	2.08299	-5.14143	-0.02411
H	0.51070	-5.97731	0.10257
C	-3.51487	-1.35316	1.53088
H	-2.67949	-0.67075	1.76220
C	-4.11934	-1.84069	2.86873
H	-4.97570	-2.51544	2.69274
H	-4.48730	-0.98537	3.46309
H	-3.38422	-2.38775	3.48013
C	-4.57527	-0.56015	0.73444
H	-4.16260	-0.16351	-0.20767
H	-4.96134	0.28516	1.33194
H	-5.44303	-1.19210	0.47752
C	-1.63798	2.66714	1.90781
C	-2.43820	3.69104	0.99857
C	-3.93071	3.35076	0.84684
H	-4.36379	4.00273	0.07165
H	-4.48538	3.51481	1.78622
H	-4.07535	2.30696	0.52774
C	-2.28192	5.16108	1.40707
H	-1.23044	5.48340	1.39194
H	-2.69168	5.33662	2.41673
H	-2.83967	5.79478	0.69905
C	-0.41552	3.28269	2.59790
H	0.19958	2.48233	3.04084
H	-0.72685	3.96240	3.40868
H	0.20766	3.84924	1.89098
C	-2.48166	1.91525	2.93773
H	-3.32830	1.38841	2.47725
H	-2.87824	2.62063	3.68848
H	-1.85606	1.17705	3.46667
B	-1.20552	2.30276	-0.42175
H	0.58164	0.95978	-1.46331
B	-0.57363	1.42538	-1.76883
C	-1.54679	0.14391	-2.21416
H	-1.83298	-0.55987	-1.39287
C	-0.32737	2.27202	-3.15096
H	0.31503	3.15729	-2.96819
C	-1.68618	2.82266	-3.66297
H	-2.04365	3.56857	-2.92822
H	-1.54934	3.37151	-4.61826
C	0.42646	1.38573	-4.17668
H	0.58220	1.93053	-5.13157
H	1.43826	1.19075	-3.76812
C	-0.24677	0.02796	-4.49290
H	0.47664	-0.62512	-5.01889
H	-1.06881	0.18316	-5.21286
C	-0.79207	-0.72700	-3.25593
H	0.06719	-1.19975	-2.73414
H	-1.43262	-1.56518	-3.60179
C	-2.89950	0.72023	-2.72010
H	-3.57531	-0.09876	-3.04239
H	-3.40110	1.20765	-1.85856
C	-2.79428	1.75937	-3.86250
H	-3.77214	2.26794	-3.97176
H	-2.63680	1.23126	-4.81872

$\mathbf{9_{Xray}}$
SCF (BP86) Energy = -1990.21225054
Enthalpy 0K = -1989.201878

Enthalpy 298K = -1989.144117
Free Energy 298K = -1989.292677
Lowest Frequency = 13.4997 cm^{-1}
Second Frequency = 19.7085 cm^{-1}
SCF (Toluene) Energy = -1990.21877452
SCF (BP86-D3BJ) Energy = -1990.50653646
SCF (BS2) Energy = -2189.93093234

```
Mg  0.06021  0.03451  0.29749
H   0.04078 -0.31055 -1.51488
H   1.75439 -2.31156 -2.21617
O   0.00729 -1.93539  1.00741
O  -0.80879 -3.78284 -0.09309
N   1.76280  1.05915  0.89468
N  -1.34628  1.35553  1.08579
C   2.84728  2.29381  2.76477
H   2.60618  2.62914  3.78392
H   3.21603  3.16448  2.19473
H   3.68357  1.57872  2.80607
C   1.62637  1.69619  2.07870
C   0.38526  1.90668  2.74332
H   0.48661  2.34350  3.74037
C  -0.95177  1.90493  2.25655
C  -1.95276  2.64210  3.13816
H  -1.94964  3.72226  2.91043
H  -1.67729  2.53318  4.19918
H  -2.98045  2.28086  2.98814
C  -2.63929  1.73856  0.55437
C  -2.79605  3.00778 -0.08125
C  -4.05639  3.35830 -0.60389
H  -4.17409  4.33161 -1.09331
C  -5.15404  2.49763 -0.51761
H  -6.12505  2.79207 -0.92922
C  -4.99240  1.25362  0.09849
H  -5.84842  0.57438  0.17369
C  -3.75427  0.85243  0.63666
C  -3.67069 -0.49642  1.34533
H  -2.60178 -0.69138  1.53397
C  -4.22752 -1.65490  0.48985
H  -5.30858 -1.53737  0.30005
H  -4.09982 -2.62039  1.01155
H  -3.71728 -1.72390 -0.48402
C  -4.38788 -0.45267  2.71583
H  -3.96770  0.32341  3.37693
H  -4.30296 -1.42352  3.23606
H  -5.46296 -0.23591  2.58670
C  -1.64816  4.00292 -0.26222
H  -0.74989  3.58670  0.22523
C  -1.33111  4.18944 -1.76329
H  -1.05938  3.23319 -2.23983
H  -0.49432  4.89665 -1.89878
H  -2.20195  4.59698 -2.30582
C  -1.94727  5.37379  0.38805
H  -2.79757  5.87661 -0.10536
H  -1.07199  6.04073  0.29997
H  -2.19667  5.28025  1.45825
C   3.05359  1.12924  0.23819
C   3.32046  2.18938 -0.67811
C   4.60517  2.29202 -1.24733
H   4.81468  3.11711 -1.93700
C   5.61300  1.36800 -0.95773
H   6.60719  1.47119 -1.40492
C   5.32450  0.29604 -0.10884
H   6.09922 -0.45105  0.09762
C   4.05854  0.14955  0.49159
C   3.81327 -1.08344  1.36039
H   2.77742 -1.02384  1.73526
C   4.75838 -1.15885  2.58129
H   5.81350 -1.24633  2.26824
H   4.52260 -2.04347  3.19911
H   4.67595 -0.26756  3.22564
C   3.94519 -2.36634  0.50623
H   3.22426 -2.37079 -0.32889
H   3.77870 -3.27071  1.11874
H   4.95606 -2.45251  0.07150
C   2.26316  3.21153 -1.09703
H   1.30547  2.91225 -0.63594
C   2.07690  3.20190 -2.63217
H   3.00529  3.49139 -3.15430
H   1.29191  3.91397 -2.93513
H   1.78914  2.20004 -2.99244
C   2.60283  4.63370 -0.59549
H   2.68538  4.67062  0.50404
H   1.82060  5.35150 -0.89945
H   3.56119  4.98727 -1.01531
C  -0.14359 -2.97770  2.07108
C  -0.32853 -4.28607  1.19528
C   0.98836 -5.02974  0.90959
H   0.79168 -5.82330  0.17140
H   1.39928 -5.49839  1.81979
H   1.74799 -4.35603  0.48347
C  -1.37153 -5.27187  1.73646
H  -2.36676 -4.81189  1.82382
H  -1.07293 -5.66169  2.72485
H  -1.45408 -6.12552  1.04495
C  -1.37123 -2.61923  2.91429
H  -1.25423 -1.60393  3.32772
H  -1.47526 -3.31961  3.75985
H  -2.29704 -2.65185  2.32356
C   1.10073 -2.96398  2.95950
H   2.02322 -3.12264  2.38599
H   1.02510 -3.75769  3.72290
H   1.17726 -1.99886  3.48742
C  -1.72349 -1.37098 -2.63093
H  -2.57221 -0.94016 -2.06025
C  -2.17420 -2.78142 -3.10003
H  -2.47939 -3.35610 -2.20347
H  -3.07819 -2.70866 -3.74062
C  -1.09753 -3.59678 -3.85836
H  -1.42681 -4.65207 -3.92830
H  -1.04059 -3.24359 -4.90260
C   0.31334 -3.55265 -3.21928
H   1.04480 -3.96717 -3.94450
H   0.31941 -4.23774 -2.35082
C   0.77902 -2.15508 -2.72522
C   1.04617 -1.14070 -3.86932
H   1.52001 -0.24612 -3.41594
H   1.79130 -1.54780 -4.58513
C  -0.20585 -0.69241 -4.66225
H   0.04932  0.20016 -5.26669
H  -0.46741 -1.47156 -5.39898
C  -1.44510 -0.37503 -3.78887
H  -1.30656  0.62768 -3.33468
H  -2.33151 -0.28541 -4.45144
B  -0.45516 -2.45865 -0.25932
B  -0.37008 -1.52337 -1.70931
```

11
SCF (BP86) Energy = -1990.22267653
Enthalpy 0K = -1989.212736
Enthalpy 298K = -1989.155789
Free Energy 298K = -1989.298964
Lowest Frequency = 23.1867 cm^{-1}
Second Frequency = 27.4747 cm^{-1}
SCF (Toluene) Energy = -1990.22744018
SCF (BP86-D3BJ) Energy = -1990.52919514

SCF (BS2) Energy = -2189.94143998

Mg	0.08706	0.03067	-0.08477
H	0.59427	1.25383	-1.50348
H	0.30126	-0.62058	-2.20113
O	-0.81587	2.00623	0.66059
O	-1.75948	4.03707	0.07784
N	1.96343	-0.32745	0.76360
N	-0.92038	-1.47292	0.98672
C	3.16705	-0.52412	2.93871
H	3.77707	0.33925	2.62959
H	3.81063	-1.41542	2.85127
H	2.89276	-0.41578	3.99963
C	1.91716	-0.65458	2.07503
C	0.76783	-1.16004	2.73961
H	0.92031	-1.35132	3.80579
C	-0.43389	-1.72599	2.21663
C	-1.11781	-2.72649	3.14086
H	-2.19345	-2.82628	2.94073
H	-0.96397	-2.44815	4.19548
H	-0.66709	-3.72564	3.00063
C	-2.06432	-2.23846	0.52836
C	-1.86325	-3.45744	-0.18260
C	-2.98767	-4.20577	-0.58542
H	-2.83118	-5.14801	-1.12224
C	-4.29023	-3.77897	-0.31490
H	-5.14917	-4.38183	-0.62784
C	-4.48095	-2.56144	0.34517
H	-5.49958	-2.20998	0.54238
C	-3.39364	-1.76958	0.76295
C	-3.68752	-0.43877	1.45563
H	-2.73500	0.11402	1.50303
C	-4.20649	-0.62995	2.90024
H	-5.14418	-1.21356	2.90410
H	-4.42000	0.34653	3.37054
H	-3.48057	-1.15979	3.53773
C	-4.69069	0.42128	0.65381
H	-4.37140	0.54774	-0.39322
H	-4.79078	1.42296	1.10888
H	-5.69900	-0.02830	0.64532
C	-0.47815	-3.99402	-0.54441
H	0.27182	-3.26524	-0.19017
C	-0.19347	-5.34504	0.15160
H	-0.27910	-5.26537	1.24863
H	0.82476	-5.69949	-0.08596
H	-0.90188	-6.12427	-0.18085
C	-0.32562	-4.12419	-2.07778
H	-1.07484	-4.81667	-2.49961
H	0.67195	-4.51488	-2.33863
H	-0.45079	-3.14672	-2.57287
C	3.26891	-0.27174	0.14381
C	3.98846	-1.48773	-0.08753
C	5.27211	-1.42813	-0.66224
H	5.81886	-2.36207	-0.83464
C	5.85818	-0.21328	-1.02873
H	6.86024	-0.18820	-1.46959
C	5.13624	0.96701	-0.83796
H	5.58234	1.92281	-1.13398
C	3.84896	0.96561	-0.26421
C	3.15757	2.31445	-0.07627
H	2.12545	2.11255	0.26214
C	3.07037	3.10241	-1.40194
H	4.07139	3.36850	-1.78371
H	2.51787	4.04804	-1.25916
H	2.55228	2.51592	-2.17717
C	3.86841	3.15562	1.01043
H	3.89611	2.63243	1.98184
H	3.36136	4.12622	1.15726
H	4.91224	3.36834	0.72041
C	3.40033	-2.87525	0.18424
H	2.41724	-2.74698	0.66765
C	3.17701	-3.61415	-1.15568
H	2.52525	-3.03280	-1.82770
H	2.71279	-4.60145	-0.98536
H	4.13445	-3.78154	-1.67961
C	4.27900	-3.73700	1.11972
H	5.26267	-3.95234	0.66665
H	3.78926	-4.70600	1.31967
H	4.46312	-3.24706	2.09058
C	-0.81059	2.84524	1.90634
C	-1.01736	4.29381	1.31165
C	-1.85939	5.22891	2.18698
H	-1.96796	6.20179	1.68109
H	-1.36980	5.40466	3.16013
H	-2.86698	4.82522	2.36506
C	0.29286	4.98685	0.90575
H	0.91366	4.32916	0.27717
H	0.88076	5.29340	1.78682
H	0.05091	5.88800	0.31989
C	0.49687	2.63736	2.66542
H	0.50149	3.27279	3.56810
H	1.37876	2.89037	2.06306
H	0.58128	1.58650	2.99055
C	-1.98677	2.37794	2.77293
H	-2.94896	2.48085	2.24636
H	-2.03575	2.96309	3.70595
H	-1.84495	1.32069	3.04496
C	-1.72449	0.64608	-1.84051
H	-2.02842	0.15788	-0.88015
C	-2.09981	2.14671	-1.67492
H	-3.20358	2.24092	-1.53479
C	-1.76551	3.02870	-2.92573
H	-2.56868	2.89134	-3.66925
H	-1.81370	4.09596	-2.63845
C	-0.39699	2.74290	-3.59661
H	0.39446	3.23569	-2.99771
H	-0.38967	3.25065	-4.58387
C	-0.00452	1.24518	-3.73727
H	1.04475	1.22339	-4.09417
C	-0.84779	0.45397	-4.76794
H	-0.38544	-0.54666	-4.88089
H	-0.80169	0.92898	-5.77020
C	-2.33122	0.26943	-4.37281
H	-2.88877	1.20008	-4.57918
H	-2.79396	-0.49585	-5.02576
C	-2.54591	-0.14513	-2.89858
H	-2.26995	-1.21226	-2.78783
H	-3.63023	-0.08964	-2.66771
B	-1.53004	2.74259	-0.32795
B	-0.12517	0.55634	-2.27519

12

SCF (BP86) Energy = -1990.21925273
Enthalpy 0K = -1989.209669
Enthalpy 298K = -1989.152602
Free Energy 298K = -1989.296050
Lowest Frequency = 23.6126 cm^{-1}
Second Frequency = 27.9983 cm^{-1}
SCF (Toluene) Energy = -1990.22368997
SCF (BP86-D3BJ) Energy = -1990.52599777
SCF (BS2) Energy = -2189.93843073

Mg	-0.06946	-0.01509	0.11238
H	-0.81332	-0.98611	1.78234
H	-0.11010	0.89366	1.96135
O	0.67661	-2.01507	-0.66207

```
O   1.76716 -4.01264 -0.23196
N  -1.89579  0.38214 -0.81639
N   1.04730  1.42211 -0.94192
C  -3.01356  0.64535 -3.02926
H  -3.69682 -0.16001 -2.71754
H  -2.70705  0.48014 -4.07423
H  -3.58958  1.58550 -3.00033
C  -1.79016  0.71746 -2.12157
C  -0.59728  1.17388 -2.74617
H  -0.70641  1.36816 -3.81737
C   0.61385  1.68686 -2.18792
C   1.38119  2.63596 -3.10128
H   0.96429  3.65542 -3.01542
H   1.27253  2.33122 -4.15448
H   2.44809  2.69840 -2.84582
C   2.19119  2.15432 -0.43256
C   3.51208  1.62436 -0.56257
C   4.60339  2.37980 -0.09135
H   5.61584  1.97809 -0.20819
C   4.42338  3.62535  0.51732
H   5.28511  4.20126  0.87077
C   3.12560  4.11527  0.68420
H   2.97561  5.07990  1.18157
C   1.99778  3.40134  0.23166
C   0.61724  4.00213  0.49838
H  -0.14079  3.28099  0.14572
C   0.39900  4.21508  2.01452
H   0.47760  3.26123  2.56195
H  -0.59907  4.64168  2.20822
H   1.14453  4.91189  2.43566
C   0.41291  5.32665 -0.27283
H   1.13454  6.09563  0.05534
H  -0.60119  5.72708 -0.09854
H   0.54331  5.19248 -1.35998
C   3.79275  0.27005 -1.21174
H   2.82116 -0.24034 -1.30879
C   4.70966 -0.62133 -0.34324
H   5.73654 -0.21952 -0.28785
H   4.78469 -1.63456 -0.77742
H   4.32989 -0.71541  0.68681
C   4.40020  0.41993 -2.62645
H   3.73457  0.97244 -3.30921
H   4.59935 -0.56969 -3.07526
H   5.36019  0.96419 -2.58187
C  -3.21655  0.36061 -0.23064
C  -3.82022 -0.86160  0.19146
C  -5.11002 -0.83415  0.75801
H  -5.57250 -1.77815  1.06665
C  -5.81468  0.36023  0.92517
H  -6.81867  0.35822  1.36233
C  -5.21009  1.55904  0.53687
H  -5.74487  2.50357  0.68740
C  -3.92209  1.59037 -0.03059
C  -3.31480  2.96479 -0.32543
H  -2.34045  2.81721 -0.82091
C  -3.06011  3.71365  1.00304
H  -4.00739  3.89859  1.53951
H  -2.58503  4.69269  0.81528
H  -2.40566  3.12984  1.67003
C  -4.19374  3.83123 -1.25655
H  -4.42108  3.32517 -2.20996
H  -3.68189  4.78067 -1.49158
H  -5.15754  4.08643 -0.78199
C  -3.15507 -2.22508  0.01309
H  -2.11704 -2.04702 -0.31984
C  -3.87885 -3.04756 -1.07994
H  -4.92850 -3.23639 -0.79448
H  -3.39477 -4.03022 -1.22662
H  -3.89062 -2.52100 -2.04973
C  -3.09437 -3.02290  1.33421
H  -2.57954 -2.44842  2.12087
H  -2.55112 -3.97403  1.18932
H  -4.10287 -3.28054  1.70199
C   0.72123 -2.76923 -1.96999
C   1.02521 -4.23475 -1.47297
C  -0.23627 -5.03092 -1.09764
H   0.06765 -5.94899 -0.57000
H  -0.81561 -5.31945 -1.99024
H  -0.89038 -4.45294 -0.42484
C   1.90715 -5.05821 -2.41772
H   2.88595 -4.58376 -2.58078
H   1.41348 -5.19917 -3.39440
H   2.08100 -6.05370 -1.97842
C   1.85755 -2.16839 -2.80329
H   1.65880 -1.09889 -2.97538
H   1.91465 -2.66488 -3.78607
H   2.83272 -2.27464 -2.30334
C  -0.59885 -2.60730 -2.71604
H  -1.46149 -2.94216 -2.12716
H  -0.56329 -3.20301 -3.64493
H  -0.74539 -1.55158 -2.99669
B   1.42263 -2.78168  0.27612
B   0.07516 -0.29345  2.33746
C   1.62388 -0.77586  1.93355
H   2.07525 -0.31951  1.02065
C   1.76370 -2.31651  1.74099
H   2.82557 -2.60156  1.91433
C   0.91271 -3.21850  2.69942
H  -0.14364 -3.23242  2.36595
H   1.28005 -4.25448  2.57837
C   0.92066 -2.84137  4.19750
H   0.67810 -3.75212  4.77909
H   1.94286 -2.55406  4.51059
C   2.46299 -0.19887  3.13081
H   3.18596 -0.95825  3.48658
H   3.06925  0.65840  2.78732
C   1.49449  0.24653  4.26908
H   1.42494  1.34973  4.27563
H   1.87268 -0.04637  5.26913
C   0.09239 -0.34765  3.96439
H  -0.68861  0.30212  4.40457
C  -0.09438 -1.74792  4.59421
H  -0.06777 -1.65224  5.70024
H  -1.10928 -2.12063  4.34709
```

13a

SCF (BP86) Energy = -1990.18238168
Enthalpy 0K = -1989.176161
Enthalpy 298K = -1989.117083
Free Energy 298K = -1989.269776
Lowest Frequency = 7.9077 cm^{-1}
Second Frequency = 16.7674 cm^{-1}
SCF (Toluene) Energy = -1990.18850308
SCF (BP86-D3BJ) Energy = -1990.47051826
SCF (BS2) Energy = -2189.90565518

```
Mg  0.18452  0.17047  0.26888
O  -0.70998  1.98077  1.05984
O  -1.47309  3.70797 -0.25796
N  -0.33827 -1.41514  1.49144
N   2.23424  0.04696  0.59882
C  -0.04077 -2.39404  3.75822
H   0.59112 -2.25586  4.64745
H   0.02661 -3.45114  3.44734
H  -1.09387 -2.22201  4.03231
C   0.39853 -1.48355  2.62062
```

```
C   1.61526 -0.77167  2.82766
H   2.01049 -0.84781  3.84416
C   2.52446 -0.19754  1.89627
C   3.91480  0.10361  2.44167
H   4.57984 -0.77059  2.33632
H   3.85991  0.34447  3.51508
H   4.38861  0.93867  1.90351
C   3.32612  0.24330 -0.32729
C   3.59184  1.52898 -0.88538
C   4.64166  1.67306 -1.81353
H   4.84468  2.66465 -2.23291
C   5.43303  0.58812 -2.19965
H   6.24717  0.72152 -2.91970
C   5.16558 -0.67155 -1.65553
H   5.77458 -1.52936 -1.96256
C   4.12453 -0.87338 -0.72890
C   3.86852 -2.30426 -0.24874
H   3.05253 -2.28111  0.49353
C   3.40698 -3.18607 -1.43147
H   2.50635 -2.77239 -1.91339
H   3.17905 -4.21084 -1.08919
H   4.19403 -3.25869 -2.20242
C   5.10672 -2.93935  0.42559
H   5.93736 -3.06256 -0.29147
H   4.85907 -3.94059  0.81909
H   5.48470 -2.32979  1.26338
C   2.82123  2.78018 -0.47283
H   1.98228  2.45037  0.16403
C   2.23096  3.54071 -1.68033
H   3.02450  3.96843 -2.31793
H   1.59769  4.37759 -1.33735
H   1.60859  2.88009 -2.30416
C   3.71701  3.71794  0.37173
H   4.08093  3.22279  1.28811
H   3.16933  4.62944  0.67144
H   4.60045  4.03968 -0.20704
C  -1.45336 -2.32555  1.33901
C  -2.75312 -1.96869  1.80523
C  -3.80762 -2.89168  1.66366
H  -4.80447 -2.61821  2.02756
C  -3.61188 -4.14231  1.07091
H  -4.44271 -4.84955  0.97835
C  -2.34455 -4.46871  0.57991
H  -2.18986 -5.43819  0.09340
C  -1.25701 -3.57969  0.68927
C   0.08303 -3.99867  0.08340
H   0.77847 -3.14671  0.18431
C  -0.07175 -4.31377 -1.42269
H  -0.76267 -5.15830 -1.58954
H   0.89951 -4.58937 -1.86594
H  -0.46876 -3.44562 -1.97528
C   0.69900 -5.20310  0.83170
H   0.86302 -4.98026  1.89969
H   1.67281 -5.48034  0.39096
H   0.04188 -6.08872  0.77240
C  -3.06364 -0.60692  2.42539
H  -2.11791 -0.04187  2.48262
C  -3.64086 -0.72126  3.85498
H  -4.61521 -1.24048  3.85634
H  -3.80177  0.28143  4.28932
H  -2.96893 -1.27675  4.53090
C  -4.03516  0.18394  1.51911
H  -3.60259  0.35795  0.51996
H  -4.28365  1.16195  1.96910
H  -4.98507 -0.36155  1.38240
C  -0.81244  3.13666  1.99097
C  -1.65231  4.17227  1.12178
C  -3.16550  4.13323  1.39563
H  -3.67515  4.76711  0.65256
H  -3.40997  4.51411  2.40144
H  -3.56517  3.11228  1.29548
C  -1.15203  5.62020  1.20023
H  -0.10341  5.71210  0.88118
H  -1.24462  6.01539  2.22646
H  -1.76400  6.25131  0.53609
C   0.61228  3.60135  2.31241
H   1.18627  2.75693  2.72857
H   0.60189  4.41084  3.06138
H   1.13506  3.96590  1.41734
C  -1.49665  2.66797  3.27744
H  -2.48817  2.23460  3.08664
H  -1.61482  3.51433  3.97597
H  -0.87304  1.90574  3.77331
B  -1.05352  2.39740 -0.27018
H   0.36633  0.95822 -1.60008
B  -0.86554  1.22707 -1.51846
C  -1.40848  1.58486 -3.01838
H  -0.94636  2.56427 -3.26668
C  -2.93628  1.85358 -3.06022
H  -3.17720  2.55875 -2.24277
H  -3.20113  2.37481 -4.00229
C  -0.90616  0.61413 -4.12296
H  -1.29123  0.94634 -5.10908
H   0.19306  0.72439 -4.17569
H  -1.39538  0.16865 -1.06318
C  -3.85807  0.60239 -2.93025
H  -4.78327  0.89993 -2.40340
H  -3.37275 -0.14437 -2.27811
C  -2.23695 -1.46440 -4.97006
H  -1.76907 -1.40628 -5.97532
H  -2.36316 -2.55342 -4.78905
C  -1.24124 -0.89493 -3.94081
H  -0.31328 -1.49080 -4.03207
H  -1.60575 -1.08133 -2.91596
C  -4.27290 -0.00332 -4.25750
H  -5.26224  0.32290 -4.61140
C  -3.60811 -0.83786 -5.08415
H  -4.13589 -1.11896 -6.00838
```

13b

SCF (BP86) Energy = -1990.17551234
Enthalpy 0K = -1989.168890
Enthalpy 298K = -1989.110043
Free Energy 298K = -1989.260801
Lowest Frequency = 13.5884 cm^{-1}
Second Frequency = 17.3542 cm^{-1}
SCF (Toluene) Energy = -1990.18111348
SCF (BP86-D3BJ) Energy = -1990.46914757
SCF (BS2) Energy = -2189.89785376

```
Mg  0.14275 -0.11991 -0.18222
O  -0.99634 -1.73603 -1.07111
O  -1.74400 -3.57491  0.09520
N  -0.32968  1.63333 -1.18359
N   2.15950 -0.13648 -0.71406
C  -0.19430  2.77272 -3.39041
H   0.41781  2.75661 -4.30360
H  -0.15786  3.78789 -2.96075
H  -1.24719  2.59387 -3.66435
C   0.29248  1.74540 -2.37781
C   1.41632  0.96646 -2.77423
H   1.71950  1.11604 -3.81390
C   2.35417  0.22726 -1.99996
C   3.66083 -0.11010 -2.70706
H   4.39669  0.70324 -2.58483
H   3.48907 -0.23444 -3.78797
```

```
H   4.11776 -1.02557 -2.30190
C   3.30478 -0.53628  0.07216
C   3.50347 -1.90152  0.43543
C   4.61175 -2.24740  1.23298
H   4.76220 -3.29868  1.50217
C   5.52430 -1.28651  1.67641
H   6.38157 -1.57597  2.29320
C   5.32331  0.05092  1.32289
H   6.02970  0.81133  1.67468
C   4.22966  0.45257  0.53154
C   4.06630  1.94852  0.25178
H   3.18924  2.08567 -0.40355
C   3.79689  2.71159  1.56875
H   2.89861  2.32850  2.08001
H   3.65305  3.78861  1.37267
H   4.64616  2.61033  2.26691
C   5.29446  2.55733 -0.46409
H   6.19289  2.52202  0.17693
H   5.10844  3.61633 -0.71433
H   5.53768  2.02532 -1.39898
C   2.59289 -3.02321 -0.05628
H   1.72938 -2.54612 -0.55047
C   2.05564 -3.90138  1.09454
H   2.86436 -4.47654  1.57882
H   1.31766 -4.62851  0.71303
H   1.56001 -3.29028  1.86525
C   3.31934 -3.89604 -1.10764
H   3.63661 -3.30562 -1.98396
H   2.66756 -4.71311 -1.46572
H   4.22196 -4.36027 -0.67310
C  -1.32818  2.62126 -0.83383
C  -2.69628  2.43885 -1.19270
C  -3.63137  3.43625 -0.85261
H  -4.68155  3.29614 -1.13256
C  -3.25040  4.59652 -0.17259
H  -3.99036  5.36594  0.07157
C  -1.91362  4.75277  0.20610
H  -1.61302  5.65148  0.75543
C  -0.94052  3.77985 -0.09610
C   0.49363  4.01125  0.38334
H   1.05152  3.07036  0.22890
C   0.53637  4.35129  1.89050
H   0.03456  5.31032  2.10727
H   1.57856  4.44279  2.23789
H   0.03979  3.57227  2.49290
C   1.19761  5.11556 -0.43924
H   1.24821  4.85838 -1.51049
H   2.23065  5.27174 -0.08115
H   0.66203  6.07738 -0.34900
C  -3.20463  1.18747 -1.90712
H  -2.33093  0.55162 -2.12880
C  -3.91628  1.51024 -3.24129
H  -4.83809  2.09477 -3.07496
H  -4.20717  0.57937 -3.75966
H  -3.27667  2.09205 -3.92645
C  -4.15122  0.38837 -0.98213
H  -3.64705  0.08201 -0.05074
H  -4.52816 -0.51547 -1.49396
H  -5.03222  0.99290 -0.70274
C  -1.29042 -2.74775 -2.12116
C  -2.12348 -3.82386 -1.29940
C  -3.64671 -3.62163 -1.37398
H  -4.12891 -4.30687 -0.65865
H  -4.04057 -3.83948 -2.38095
H  -3.92880 -2.59427 -1.09616
C  -1.77946 -5.28153 -1.62996
H  -0.71612 -5.50340 -1.45690
H  -2.02261 -5.51626 -2.68057
H  -2.37359 -5.94951 -0.98592
C   0.04911 -3.26962 -2.65297
H   0.65149 -2.42137 -3.01830
H  -0.10643 -3.96691 -3.49320
H   0.62276 -3.79038 -1.87374
C  -2.05814 -2.06794 -3.25728
H  -2.98307 -1.59025 -2.90572
H  -2.32097 -2.80682 -4.03406
H  -1.42389 -1.29731 -3.72597
B  -1.22000 -2.30777  0.22805
H   0.46871 -1.18166  1.50846
B  -0.78801 -1.32931  1.57749
C  -1.09802 -1.87427  3.09455
H  -0.44974 -2.77682  3.13698
C  -2.51975 -2.46021  3.34304
H  -2.83314 -2.99429  2.42761
H  -2.45672 -3.23587  4.13467
C  -0.54199 -0.98841  4.24959
H  -0.37621 -1.62435  5.14464
H   0.46763 -0.65596  3.93678
C  -1.31190  0.26180  4.75435
H  -0.60339  0.83195  5.38674
H  -2.12291 -0.03245  5.44212
C  -1.87898  1.24475  3.71061
H  -1.08302  1.49670  2.97771
H  -2.09614  2.20583  4.22333
H  -1.26271 -0.18917  1.31684
C  -3.66603 -1.50369  3.77886
H  -4.60622 -2.08652  3.76258
H  -3.51831 -1.22902  4.83661
C  -3.86257 -0.25504  2.94360
H  -4.72043 -0.27604  2.25595
C  -3.11736  0.87053  2.92819
H  -3.43887  1.66074  2.23294
```

13c

SCF (BP86) Energy = -1990.17452978
Enthalpy 0K = -1989.167970
Enthalpy 298K = -1989.109029
Free Energy 298K = -1989.260107
Lowest Frequency = 15.9939 cm^{-1}
Second Frequency = 19.6725 cm^{-1}
SCF (Toluene) Energy = -1990.18025624
SCF (BP86-D3BJ) Energy = -1990.46812491
SCF (BS2) Energy = -2189.89709474

```
Mg -0.22188  0.12616  0.23879
O  -0.35070  2.23573  0.77797
O   0.35024  3.95470 -0.58503
N  -1.95828 -0.88902  0.75930
N   1.04442 -0.98304  1.46592
C  -3.32358 -1.79790  2.63146
H  -3.24503 -1.94703  3.71813
H  -3.59103 -2.76417  2.16925
H  -4.16135 -1.11505  2.41921
C  -2.01497 -1.28164  2.04979
C  -0.89902 -1.29100  2.93328
H  -1.15089 -1.52483  3.97100
C   0.49594 -1.29511  2.66196
C   1.38330 -1.73745  3.81855
H   1.55988 -2.82631  3.78539
H   0.89967 -1.51213  4.78179
H   2.36986 -1.25093  3.78262
C   2.41051 -1.38699  1.21490
C   3.46832 -0.43138  1.23206
C   4.78147 -0.85194  0.94493
H   5.59099 -0.11391  0.96603
C   5.07361 -2.18580  0.64580
```

```
H   6.10116 -2.49473  0.42715
C   4.03312 -3.11996  0.63033
H   4.25471 -4.16656  0.39257
C   2.70205 -2.75203  0.90794
C   1.62869 -3.83996  0.82486
H   0.65715 -3.38583  1.08487
C   1.52645 -4.38882 -0.61627
H   1.30795 -3.58617 -1.33940
H   0.72865 -5.14881 -0.68786
H   2.47121 -4.86855 -0.92676
C   1.88868 -5.00387  1.80960
H   2.81989 -5.54177  1.55883
H   1.06240 -5.73487  1.76854
H   1.98110 -4.65571  2.85191
C   3.23930  1.03128  1.60081
H   2.14986  1.17121  1.70361
C   3.74374  1.99370  0.50414
H   4.84064  1.93375  0.38973
H   3.50434  3.04154  0.76072
H   3.28767  1.76366 -0.47192
C   3.88958  1.37002  2.96317
H   3.50161  0.73118  3.77465
H   3.70112  2.42243  3.24069
H   4.98395  1.22883  2.92245
C  -3.11982 -1.12348 -0.07237
C  -4.15424 -0.14733 -0.17356
C  -5.29368 -0.43536 -0.95011
H  -6.09239  0.31199 -1.01809
C  -5.42752 -1.64758 -1.63295
H  -6.32636 -1.85744 -2.22219
C  -4.38662 -2.57847 -1.57056
H  -4.47256 -3.51910 -2.12592
C  -3.22337 -2.33781 -0.81305
C  -2.11126 -3.38682 -0.84408
H  -1.25341 -2.98376 -0.27717
C  -1.64611 -3.64337 -2.29642
H  -2.46348 -4.05094 -2.91639
H  -0.81898 -4.37189 -2.31945
H  -1.29856 -2.71224 -2.77459
C  -2.54418 -4.70775 -0.16802
H  -2.83116 -4.55219  0.88577
H  -1.72205 -5.44476 -0.18892
H  -3.40888 -5.15770 -0.68725
C  -4.06592  1.22310  0.49678
H  -3.12015  1.25720  1.06417
C  -5.22601  1.48290  1.48497
H  -6.20110  1.49706  0.96722
H  -5.10112  2.46242  1.97984
H  -5.28014  0.71301  2.27297
C  -4.01994  2.33530 -0.57651
H  -3.14183  2.22199 -1.23380
H  -3.98234  3.33438 -0.10638
H  -4.92106  2.31145 -1.21367
C  -0.38302  3.43403  1.66008
C  -0.17133  4.61874  0.61189
C  -1.47310  5.31690  0.18230
H  -1.24274  6.02143 -0.63278
H  -1.92745  5.88448  1.01176
H  -2.20984  4.59399 -0.19953
C   0.85503  5.67491  1.04404
H   1.84474  5.23464  1.23510
H   0.52206  6.20467  1.95301
H   0.96710  6.41834  0.23865
C   0.74966  3.29746  2.68354
H   0.62007  2.36266  3.25319
H   0.73233  4.13699  3.39864
H   1.73691  3.27467  2.20168
C  -1.72575  3.45633  2.39618
H  -2.57874  3.46729  1.70401
H  -1.79188  4.34705  3.04449
H  -1.81175  2.56500  3.03955
B   0.12957  2.59845 -0.52590
H  -0.85182  0.72456 -1.59145
B   0.25796  1.33343 -1.68825
C   0.41180  1.76884 -3.26483
H  -0.42841  2.48821 -3.37399
H   1.12296  0.51709 -1.27695
C   1.84020 -1.14299 -3.54617
H   2.21213 -2.11992 -3.92081
H   1.14473 -1.40951 -2.72139
C   1.04959 -0.46375 -4.68185
H   0.45579 -1.25086 -5.18631
H   1.76619 -0.12202 -5.44833
C   0.05486  0.67108 -4.31202
H  -0.87737  0.19218 -3.95364
H  -0.21936  1.16309 -5.26911
C   1.66395  2.62698 -3.61547
H   1.40291  3.32811 -4.43533
H   1.90467  3.26464 -2.74472
C   2.95794  1.90179 -4.07891
H   3.74061  2.67332 -4.20541
H   2.79147  1.49387 -5.09017
C   3.02373 -0.42190 -2.94317
H   3.57044 -1.02927 -2.20630
C   3.49685  0.82421 -3.15875
H   4.38633  1.10583 -2.57751
```

13d

SCF (BP86) Energy = -1990.15965826
Enthalpy 0K = -1989.154360
Enthalpy 298K = -1989.095038
Free Energy 298K = -1989.248192
Lowest Frequency = 10.5504 cm^{-1}
Second Frequency = 16.6256 cm^{-1}
SCF (Toluene) Energy = -1990.16574614
SCF (BP86-D3BJ) Energy = -1990.45012874
SCF (BS2) Energy = -2189.88263520

```
Mg -0.23516  0.09985  0.27713
O  -0.26523  2.21258  0.80034
O   0.54014  3.90391 -0.53936
N  -2.07178 -0.79109  0.67892
N   0.88337 -1.12517  1.53331
C  -3.58973 -1.68323  2.43889
H  -3.57643 -1.88541  3.51968
H  -3.89515 -2.60796  1.91881
H  -4.36984 -0.93737  2.21964
C  -2.22225 -1.23112  1.94580
C  -1.15718 -1.35435  2.88265
H  -1.48022 -1.61273  3.89429
C   0.24603 -1.44660  2.68383
C   1.03671 -2.00278  3.86115
H   1.21561 -3.08478  3.73879
H   0.48289 -1.86145  4.80178
H   2.02598 -1.52712  3.94529
C   2.23082 -1.62376  1.35167
C   3.35808 -0.76766  1.51978
C   4.65289 -1.28689  1.32443
H   5.51587 -0.62595  1.46221
C   4.85961 -2.62272  0.97013
H   5.87390 -3.00919  0.82575
C   3.75090 -3.45656  0.79724
H   3.90530 -4.50272  0.50971
C   2.43511 -2.98752  0.97757
C   1.28664 -3.96594  0.72148
H   0.33702 -3.44066  0.92112
```

C	1.28185	-4.41339	-0.75795
H	1.18903	-3.55266	-1.44041
H	0.44249	-5.10367	-0.95256
H	2.21482	-4.94432	-1.01598
C	1.34563	-5.20475	1.64513
H	2.25320	-5.80490	1.45685
H	0.47342	-5.85802	1.46829
H	1.34852	-4.92842	2.71283
C	3.21890	0.69210	1.94118
H	2.13942	0.91663	1.97393
C	3.87844	1.64477	0.92088
H	4.96549	1.46838	0.84651
H	3.74340	2.70009	1.21990
H	3.44679	1.51481	-0.08416
C	3.79370	0.93451	3.35643
H	3.30957	0.29387	4.11272
H	3.65213	1.98632	3.66269
H	4.87700	0.72293	3.38513
C	-3.20319	-0.91481	-0.21722
C	-4.17477	0.12408	-0.31605
C	-5.29351	-0.06166	-1.15173
H	-6.04467	0.73363	-1.21741
C	-5.46600	-1.23219	-1.89565
H	-6.34881	-1.36274	-2.53025
C	-4.48247	-2.22346	-1.83519
H	-4.59631	-3.13129	-2.43797
C	-3.34185	-2.08553	-1.01984
C	-2.29034	-3.19469	-1.05673
H	-1.43501	-2.86651	-0.44011
C	-1.78176	-3.41422	-2.50049
H	-2.59498	-3.74474	-3.16980
H	-0.99813	-4.18914	-2.52512
H	-1.35982	-2.48581	-2.92023
C	-2.82350	-4.51566	-0.45667
H	-3.14214	-4.38850	0.59188
H	-2.04423	-5.29776	-0.48051
H	-3.69128	-4.89175	-1.02696
C	-4.03769	1.45793	0.41693
H	-3.11963	1.41118	1.02727
C	-5.22584	1.75069	1.36153
H	-6.17208	1.84845	0.80113
H	-5.06434	2.69917	1.90394
H	-5.36559	0.95452	2.11189
C	-3.87562	2.60591	-0.60649
H	-2.97208	2.46911	-1.22374
H	-3.80960	3.58320	-0.09553
H	-4.74195	2.65601	-1.28858
C	-0.24800	3.40330	1.69449
C	0.06152	4.58542	0.66551
C	-1.17829	5.39105	0.24155
H	-0.88638	6.09577	-0.55338
H	-1.59578	5.97150	1.08137
H	-1.96471	4.73723	-0.16434
C	1.16521	5.55143	1.11927
H	2.11586	5.03189	1.30961
H	0.86896	6.09426	2.03312
H	1.34122	6.29380	0.32448
C	0.84701	3.18752	2.74489
H	0.63651	2.26630	3.31251
H	0.87222	4.02735	3.45927
H	1.84156	3.09299	2.28733
C	-1.60467	3.49941	2.39860
H	-2.43827	3.57500	1.68749
H	-1.63027	4.38250	3.06000
H	-1.76219	2.60586	3.02516
B	0.25318	2.56051	-0.49339
H	-0.68106	0.63585	-1.58702
B	0.40895	1.28322	-1.63498

C	0.64726	1.63036	-3.21658
H	-0.28186	2.17142	-3.49975
H	1.27481	0.49614	-1.15583
C	0.58659	0.37415	-4.17219
H	-0.47258	0.05008	-4.14119
H	0.75870	0.70827	-5.21710
C	1.74178	2.71008	-3.51319
H	1.81292	2.82478	-4.61425
H	1.37794	3.67876	-3.11996
C	1.42586	-0.91519	-3.91043
H	1.52159	-1.04954	-2.81669
H	0.85581	-1.79196	-4.27813
C	3.19543	2.48095	-2.98168
H	3.88929	3.13906	-3.53947
H	3.26561	2.77340	-1.91931
C	3.49972	1.01784	-3.16568
H	3.38009	0.38043	-2.27717
C	3.45524	0.42689	-4.37451
H	3.50399	1.06005	-5.27309
C	2.85895	-0.95215	-4.53582
H	2.80348	-1.22562	-5.60522
H	3.45469	-1.74249	-4.03979

Single Crystal X-ray Diffraction Analysis

Data were collected for compounds **9 - 12** on a SuperNova, Dual Cu at zero, EosS2 diffractometer. The crystals were all kept at 150(2) K during data collection. Using Olex2,[9] the structures were solved via the olex2.solve routine and refined with the ShelXL[10] refinement package using Least Squares minimization. For compound **9**, H2 and H40 (attached to B2 and C40, respectively) were located and refined without restraints. From the raw data for compound **11** it was evident that the diffraction pattern resulted from a twin – wherein the second component was small. The twin angle was determined to be in the region of 178°and, as such, most reflections were overlapped. Data integration to account for the twin was carried with as small a mask as possible in an effort to maximize the quality of the arising data sets. The *R*(int) for the data extracted pertaining to the minor component was poor and, after parallel refinements, the results presented here are based solely on the data arising from the major component in the sample. Overall, we have here an unambiguous characterization of this material, and a credible convergence. Residual electron density is in the region of C40-C42, for which some soft ADP restraints were included in the model. There is either some minor disorder in this region – or the maxima are artifacts of the crystal quality/twinning. In any event, it was not possible to attain a chemically sensible disorder model for this region with an accompanying enhancement of the residuals. H2a and H2b were located and refined freely, subject to being equidistant from B2. For **12** the hydrogens attached to B2 and C36 were located and refined without restraints. A further crystal resulting from a toluene solution of a mixture of **11** and **12** was found to represent an average of these two structural isomers. In particular, C38-C42 were each found to be disordered in a 78:22 ratio. ADP restraints were applied to the fractional occupancy carbons in the final least squares. H36 was located and refined subject to being a distance of 0.98 Å from C36, while H2A and H2A (attached to B2) were located and refined freely.

.

Table S1: Single crystal X-ray diffraction analysis of compounds **9** – **12** and the 78:22 co-crystal of **11** and **12**.

Compound	**9**	**10**	**11**	**12**	**11/12**
Empirical formula	$C_{43}H_{68}B_2MgN_2O_2$	$C_{53}H_{68}B_2MgN_2O_2$	$C_{43}H_{68}B_2MgN_2O_2$	$C_{43}H_{68}B_2MgN_2O_2$	$C_{43}H_{68}B_2MgN_2O_2$
Formula weight	690.92	811.02	690.92	690.92	690.92
Temperature/K	150.00(10)	150.00(10)	150.01(10)	298.15	150.00(10)
Crystal system	monoclinic	monoclinic	monoclinic	monoclinic	monoclinic
Space group	$P2_1/n$	$P2_1$	$P2_1/n$	$P2_1/n$	$P2_1/n$
a/Å	10.4806(1)	12.1210(7)	9.9486(2)	9.9657(1)	9.94853(16)
b/Å	20.9444(1)	16.8031(8)	21.5144(4)	21.5829(2)	21.5169(5)
c/Å	19.3236(1)	12.9376(9)	19.0011(3)	18.8992(2)	18.9880(4)
α/°	90	90	90	90	90
β/°	100.418(1)	116.747(8)	92.140(2)	92.242(1)	92.0642(18)
γ/°	90	90	90	90	90
U/Å^3	4171.79(5)	2353.1(3)	4064.12(13)	4061.89(7)	4061.95(14)
Z	4	2	4	4	4
ρ_{calc}g/cm^3	1.100	1.145	1.129	1.130	1.130
μ/mm^{-1}	0.629	0.634	0.645	0.646	0.646
F(000)	1512.0	876.0	1512.0	1512.0	1512.0
Crystal size/mm^3	0.313 × 0.124 × 0.122	0.08 × 0.058 × 0.043	0.187 × 0.127 × 0.088	0.284 × 0.239 × 0.182	0.21 × 0.08 × 0.065
2θ range for data collection/°	6.28 to 146.26	7.652 to 147.346	6.208 to 144.258	6.218 to 146.896	6.21 to 146.59
Index ranges	-10 ≤ h ≤ 13, -25 ≤ k ≤ 25, -23 ≤ l ≤ 23	-15 ≤ h ≤ 11, -20 ≤ k ≤ 16, -12 ≤ l ≤ 15	-12 ≤ h ≤ 12, -25 ≤ k ≤ 26, -23 ≤ l ≤ 23	-12 ≤ h ≤ 11, -24 ≤ k ≤ 26, -23 ≤ l ≤ 23	-9 ≤ h ≤ 12, -25 ≤ k ≤ 26, -21 ≤ l ≤ 23
Reflections collected	48301	16025	55552	55827	30529
Independent reflections, R_{int}	8324 [R_{int}= 0.0290, R_{sigma}= 0.0175]	8292 [R_{int} = 0.0533, R_{sigma} = 0.0927]	8018 [R_{int} = 0.0897, R_{sigma} = 0.0536]	8171 [R_{int} = 0.0510, R_{sigma} = 0.0280]	8072 [R_{int} = 0.0495, R_{sigma} = 0.0451]
Data/restraints/parameters	8324/0/473	8292/1/555	8018/25/473	8171/0/477	8072/49/522
Goodness-of-fit on F^2	1.024	1.035	1.050	1.038	1.033
Final R1, wR_2[I>=2σ (I)]	R_1 = 0.0372, wR_2 = 0.0958	R_1 = 0.0547, wR_2 = 0.0950	R_1 = 0.0855, wR_2 = 0.2399	R_1 = 0.0490, wR_2 = 0.1279	R_1 = 0.0555, wR_2 = 0.1359
Final R1, wR_2[all data]	R_1 = 0.0407, wR_2 = 0.0984	R_1 = 0.0788, wR_2 = 0.1049	R_1 = 0.1081, wR_2 = 0.2567	R_1 = 0.0561, wR_2 = 0.1335	R_1 = 0.0742, wR_2 = 0.1464
Largest diff. peak/hole / e Å^{-3}	0.32/-0.26	0.20/-0.20	0.97/-0.30	0.61/-0.32	0.38/-0.34
Flack Parameter		-0.06(6)			

References

[1] Dove, A. P., Gibson, V. C., Hormnirun, P., Marshall, E. L., Segal, J. A., White, A. J. P., Williams, D. J. *Dalton. Trans.* 3088 (2003).

[2] Pécharman, A. F.; Colebatch, A.; Hill, M. S.; McMullin, C. L.; Mahon, M. F.; Weetman, C. *Nature Commun.* **8**, 15022 (2017).

[3] Frisch, M. J.; Trucks, G. W.; Schlegel, H. B.; Scuseria, G. E.; Robb, M. A.; Cheeseman, J. R.; Scalmani, G.; Barone, V.; Mennucci, B.; Petersson, G. A.; Nakatsuji, H.; Caricato, M.; Li, X.; Hratchian, H. P.; Izmaylov, A. F.; Bloino, J.; Zheng, G.; Sonnenberg, J. L.; Hada, M.; Ehara, M.; Toyota, K.; Fukuda, R.; Hasegawa, J.; Ishida, M.; Nakajima, T.; Honda, Y.; Kitao, O.; Nakai, H.; Vreven, T.; Montgomery, J. A., Jr.; Peralta, J. E.; Ogliaro, F.; Bearpark, M.; Heyd, J. J.; Brothers, E.; Kudin, K. N.; Staroverov, V. N.; Kobayashi, R.; Normand, J.; Raghavachari, K.; Rendell, A.; Burant, J. C.; Iyengar, S. S.; Tomasi, J.; Cossi, M.; Rega, N.; Millam, J. M.; Klene, M.; Knox, J. E.; Cross, J. B.; Bakken, V.; Adamo, C.; Jaramillo, J.; Gomperts, R.; Stratmann, R. E.; Yazyev, O.; Austin, A. J.; Cammi, R.; Pomelli, C.; Ochterski, J. W.; Martin, R. L.; Morokuma, K.; Zakrzewski, V. G.; Voth, G. A.; Salvador, P.; Dannenberg, J. J.; Dapprich, S.; Daniels, A. D.; Farkas, O.; Foresman, J. B.; Ortiz, J. V.; Cioslowski, J.; Fox, D. J. Gaussian 09 (Revision D.01); Gaussian Inc.: Wallingford, CT, 2009.

[4] Andrae, D., Häußermann, U., Dolg, M., Stoll, H., Preuß, H., *Theor. Chim. Acta* **77**, 123−141 (1990).

[5] (a) Hariharan, P. C., Pople, J. A. *Theor. Chim. Acta* **28**, 213−222 (1973). (b) Hehre, W. J., Ditchfield, R., Pople, J. A. *J. Chem. Phys.* **56**, 2257 (1972).

[6] (a) Becke, A. D. *Phys. Rev. A: At., Mol., Opt. Phys.* **38**, 3098 (1988). (b) Perdew, J. P. *Phys. Rev. B: Condens. Matter Mater. Phys.* **33**, 8822−8824 (1986).

[7] Tomasi, J., Mennucci, B., Cammi, R. *Chem. Rev.* **105**, 2999−3094 (2005).

[8] S. Grimme, S. Ehrlich and L. Goerigk, "Effect of the damping function in dispersion corrected density functional theory," *J. Comp. Chem.* **32**, 1456-1465 (2011).

[9] Bourhis, L. J., Dolomanov, O. V., Gildea, R. J., Howard, J. A. K., Puschmann, H. *Acta Cryst. A* **71**, 59-75 (2015).

[10] Sheldrick, G. M. *Acta Cryst. C***71**, 3-8 (2015).
